# Early neural specification of stem cells is mediated by a set of SOX2-dependent neural-associated enhancers

**DOI:** 10.1016/j.stemcr.2024.03.003

**Published:** 2024-04-04

**Authors:** Pavel Tsaytler, Gaby Blaess, Manuela Scholze-Wittler, Frederic Koch, Bernhard G. Herrmann

**Affiliations:** 1 Department of Developmental Genetics, Max Planck Institute for Molecular Genetics, 14195 Berlin, Germany

**Keywords:** Sox2, enhancers, embryonic stem cells, neural induction, default neurogenesis, mesoderm, differentiation, lineage choice

## Abstract

SOX2 is a transcription factor involved in the regulatory network maintaining the pluripotency of embryonic stem cells in culture as well as in early embryos. In addition, SOX2 plays a pivotal role in neural stem cell formation and neurogenesis. How SOX2 can serve both processes has remained elusive. Here, we identified a set of SOX2-dependent neural-associated enhancers required for neural lineage priming. They form a distinct subgroup (1,898) among 8,531 OCT4/SOX2/NANOG-bound enhancers characterized by enhanced SOX2 binding and chromatin accessibility. Activation of these enhancers is triggered by neural induction of wild-type cells or by default in *Smad4*-ablated cells resistant to mesoderm induction and is antagonized by mesodermal transcription factors via *Sox2* repression. Our data provide mechanistic insight into the transition from the pluripotency state to the early neural fate and into the regulation of early neural versus mesodermal specification in embryonic stem cells and embryos.

## Highlights

• Early neural specification is mediated by a set of SOX2-dependent enhancers (SONAEs)
• SONAEs constitute a distinct subgroup of OCT4/SOX2/NANOG-bound stem cell enhancers
• SONAEs are transiently active and replaced by neural progenitor cell enhancers
• Neural specification is antagonized by mesodermal factors via *Sox2* repression

## Graphical abstract

**Figure:**
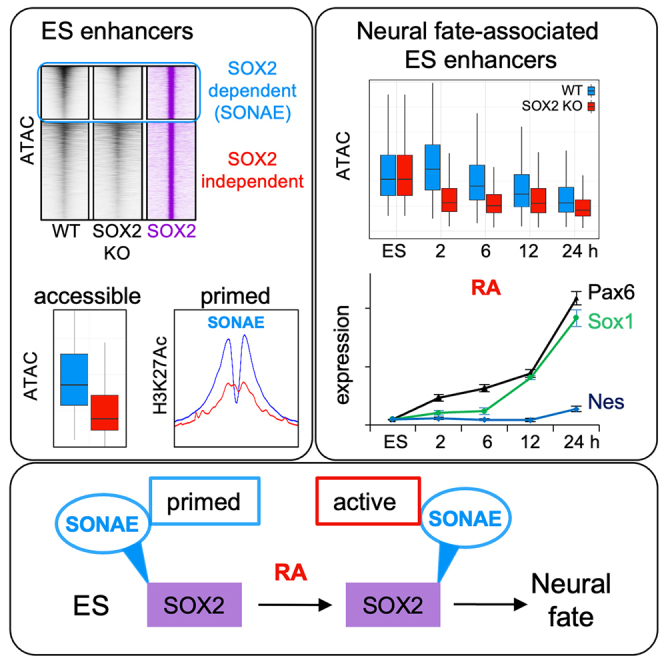

## Teaser

The authors identified a subset of OCT4/SOX2/NANOG-bound stem cell enhancers that are SOX2 dependent and activated immediately following neural induction by RA or by default. They transiently control early neural fate genes and are replaced by neural progenitor enhancers continuing the neural program. The data provide mechanistic insight into the switch from pluripotency to the neural fate.

## Introduction

OCT4, SOX2, and NANOG are the core pluripotency transcription factors (TFs) that orchestrate a gene regulatory network maintaining the undifferentiated state of embryonic stem cells (ESCs) and repress lineage-specific genes (Masui et al., 2007). SOX2, essential for pluripotency maintenance in mouse epiblast cells (Avilion et al., 2003), is also critical for neuroectoderm (NE) specification (Thomson et al., 2011; Bergsland et al., 2011; Zhang and Cui, 2014; Zhang et al., 2019; Bunina et al., 2020). SOX2 contributes to NE specification by repressing TFs regulating other lineages, e.g., mesoderm (ME), but which enhancer elements are critical for this function remains obscure (Wang et al., 2012; Zhang and Cui, 2014).

In ESCs, OCT4 and SOX2 co-localize and cooperate at thousands of loci (Chen et al., 2008; Whyte et al., 2013; Dodonova et al., 2020). *Oct4* depletion reduces accessibility levels at enhancers associated with pluripotency genes but not at all OCT4/SOX2/NANOG-bound enhancers (OSNs) (King and Klose, 2017; Xiong et al., 2022). Likewise, SOX2 promotes chromatin opening of a fraction of OSNs (Strebinger et al., 2019; Maresca et al., 2023). Thus, SOX2 and OCT4 selectively regulate the accessibility of a subset of their binding sites (Friman et al., 2019). In caudal epiblast-like cells (EpiLCs), SOX2 binds to regions associated with pluripotency as well as neural fate genes, and the accessibility of these regions correlates with the *Sox2* levels (Blassberg et al., 2022).

In this study, we investigated the role of SOX2-mediated chromatin accessibility at enhancers in differentiating ESCs. We found a large set of OSNs that depend on SOX2 for chromatin opening. SOX2-opened OSNs are highly accessible and strongly associated with neural fate genes. We show that these enhancers become transiently activated immediately following neural induction and are required for up-regulation of neural genes, including *Pax6*, and for neural lineage priming. In contrast, they undergo rapid closure and inactivation during mesodermal differentiation. Our results provide mechanistic insight into the role of SOX2 in the regulation of early neural versus mesodermal induction in ESCs and embryos.

## Results

### Rapid SOX2 protein degradation in ESCs identifies a set of SOX2-dependent putative enhancers

**Figure 1:**
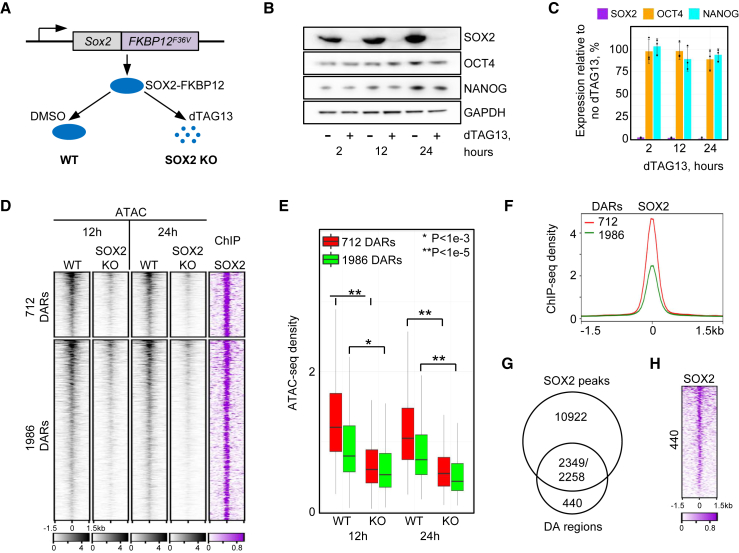
SOX2-dependent DARs identified in mouse ESCs (A) Schematic of SOX2 degradation by the dTAG system. (B) Western blot analysis of dTAG13-treated cells at indicated time points. (C) Bar plot showing protein expression levels in dTAG-treated cells. The data in (B) and (C) show representative results from three independent experiments. Data are mean + SEM. (D) Clustered heatmaps of ATAC-seq and ChIP-seq reads in ESCs centered on DARs. Each ATAC-seq sample contains merged data from two independent experiments (see Figure S1A). (E) Boxplot of normalized ATAC-seq density in clusters defined in (D). p values: paired two-tailed Student’s t test. Boxplots show median values and first to third interquartile ranges; whiskers: 1.5× the interquartile ranges. (F) Average SOX2 ChIP-seq density profiles in clusters defined in (D). (G) Venn diagram of SOX2 ChIP-seq peaks overlapping with DARs. (H) Heatmap showing SOX2 ChIP-seq reads in 440 regions defined in (G). See also Figure S1.

To investigate the role of SOX2 in establishing enhancer accessibility, we employed a dTAG system to generate dTAG13-inducible SOX2 knockout (KO) cells (SOX2-FKBP12) (Figure 1A) (Nabet et al., 2018). dTAG13 administration eliminated SOX2 at 2 h after treatment, and SOX2 loss persisted for at least 24 h (Figure 1B). SOX2 protein was basically absent at 2, 12, and 24 h (1.5%, 1.2%, and 0.3% of the wild-type [WT] levels) in SOX2 KO cells (Figures 1B and 1C). OCT4 and NANOG levels remained largely unaffected in SOX2 KO, suggesting that SOX2-FKBP12/dTAG13 is a suitable model to selectively study effects of SOX2 depletion (Figures 1B and 1C).

To assess the effect of SOX2 ablation on chromatin accessibility, we investigated accessibility changes after 12 and 24 h using Assay for Transposase Accessible Chromatin with sequencing (ATAC-seq). We identified 784 or 3,001 differentially accessible regions (DARs) between WT and SOX2 KO cells at 12 or 24 h, respectively (Figures S1A and S1B). Over 96% of the DARs were located outside of gene promoters, suggesting strong enrichment of putative enhancers (Figure S1B). The majority of DARs showed reduced accessibility in SOX2 KO cells, suggesting that SOX2 mostly regulates opening of chromatin (Figure S1C). We will refer to the DARs with reduced or increased accessibility in SOX2 KO cells as SOX2-opened or SOX2-closed regions, respectively.

To assess whether accessibility changes at DARs are directly caused by SOX2, we monitored the SOX2 occupancy using a public chromatin immunoprecipitation sequencing (ChIP-seq) dataset (Whyte et al., 2013). SOX2 was enriched at SOX2-opened, but not at SOX2-closed, sites (Figure S1C). Furthermore, at 12 h, we detected only 10 closed sites compared to 303 sites at 24 h. This suggests that SOX2-closed sites are not regulated by SOX2 directly (Figure S1C). During development, SOX2 antagonizes and inhibits expression of the ME TFs *Eomes* and *T* (Koch et al., 2017; Blassberg et al., 2022; Wang et al., 2012; Thomson et al., 2011). We recently showed that ME TFs induce differentiation by activating ME enhancers via increasing their accessibility (Tsaytler et al., 2023). We hypothesized that opening of DARs in SOX2 KO cells may be mediated by ME TFs and analyzed the binding of pSMAD1/5, pSMAD2, EOMES, and T to these DARs in ME cells (Figures S1D and S1E). 177 of 303 SOX2-closed regions (58%) were bound by ME TFs. Therefore, these sites are regulated by ME-specific TFs, which activate enhancers at mesodermal lineage genes, such as *Tbx20* or *Tpm1*, in the absence of *Sox2* (Figure S1F).

For SOX2-opened enhancers, we identified 774 DARs at 12 h and 2,698 DARs at 24 h, 712 of which are common (Figures S1C and S1G). Further quantification of ATAC-seq density showed the closure of all 2,698 DARs at 12 h (Figure 1D). However, the 712 common DARs exhibited higher accessibility and stronger SOX2 binding in WT ESCs than the other 1,986 DARs (Figures 1E and 1F), resulting in more significant accessibility reduction at 12 h of SOX2 depletion (Figures 1D and 1E). We further analyzed all 2,698 SOX2-dependent regions whose accessibility was reduced already at 12 h. 2,349 SOX2 peaks were detected in 2,258 out of 2,698 (over 83%) of these sites (Figures 1G and S1C). Moreover, the rest of the regions (440) also displayed SOX2 binding at lower signal intensity (Figure 1H). Therefore, accessibility of SOX2-opened sites in ESCs is mediated via direct SOX2 binding. We compared our data with other ATAC-seq datasets derived from murine *Sox2* KO cells (Friman et al., 2019; Blassberg et al., 2022, Maresca et al., 2023). The accessibility of the SOX2-opened regions identified here was also strongly reduced upon *Sox2* ablation in these datasets, confirming our approach and validating our findings in ESCs (Figures S1H–S1J).

### SOX2-dependent DARs are OSN enhancers associated with neural development genes

**Figure 2:**
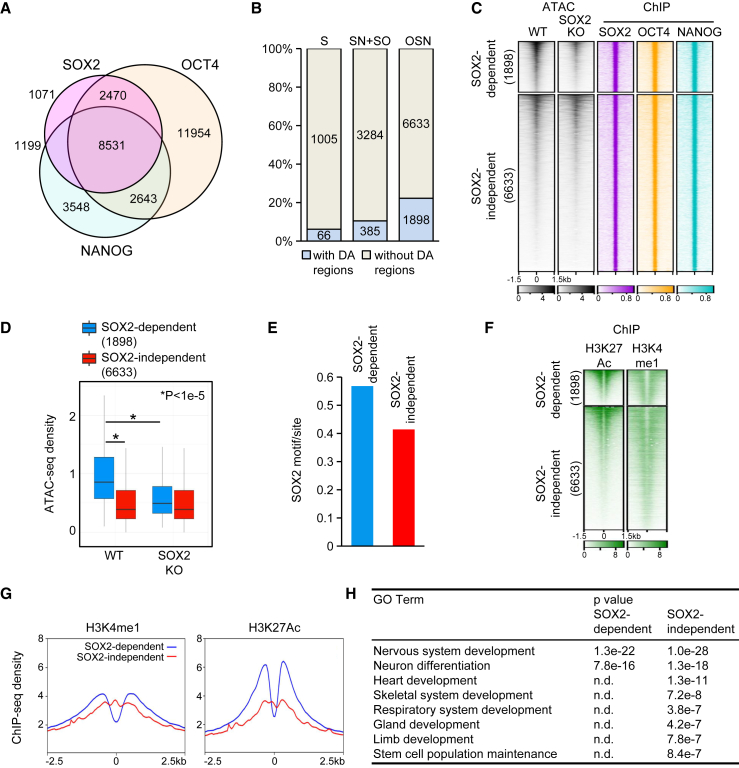
SOX2-dependent OSN enhancers are highly accessible and associated with neural development genes (A) Venn diagram of ChIP-seq peaks in ESCs. (B) Bar plot of SOX2 (S), co-bound SOX2/NANOG and SOX2/OCT4 (SN + SO), and OCT4/SOX2/NANOG (OSN) peaks listed in (A). (C) Clustered heatmaps of SOX2-dependent or -independent OSN peaks. Each ATAC-seq sample contains merged data from two independent experiments. (D) Boxplot of normalized ATAC-seq density. p value: paired two-tailed Student’s t test. Boxplots show median values and first to third interquartile ranges; whiskers: 1.5× the interquartile ranges. (E) Bar plot of SOX2 motif occurrence in clustered regions displayed in (C). (F) Heatmaps of histone modification enrichments in clustered regions identified in (C). (G) Average ChIP-seq density profiles of histone modifications from (F). (H) GO term enrichment among genes associated with regions from clusters defined in (C). See also Figure S2.

Above, we showed that 2,349 SOX2 peaks overlapped with SOX2-opened sites (Figure 1G). However, the accessibility of the remaining 10,922 peaks was not altered by SOX2 KO (Figures S2A and S2B). Therefore, accessibility of only a fraction of SOX2 peaks is SOX2 dependent, at least within 24 h of SOX2 ablation. One would expect that SOX2 dependency occurs at enhancers not bound by other pluripotency TFs, such as OCT4 and NANOG, which co-localize with SOX2 on many sites (Figure 2A) (Chen et al., 2008; Whyte et al., 2013). However, we observed the opposite. Only 66 (6%) of the unique SOX2 peaks represented DARs, whereas 1,898 (22%) of the OSNs and 385 (10.5%) of peaks co-bound by NANOG (SOX2/NANOG) or OCT4 (SOX2/OCT4) were SOX2 dependent (Figure 2B). Therefore, selective SOX2 sensitivity is a property of a subset of OSNs.

Since OCT4, SOX2, and NANOG co-occupancy is strongly associated with enhancer activity, we focused on OCT4/SOX2/NANOG-co-bound SOX2-opened regions (Chen et al., 2008). We set out to identify features that differentiate the 1,898 SOX2-dependent OSNs from the remaining 6,633 SOX2-independent OSNs (Figure 2C). SOX2-dependent OSNs displayed a significantly greater accessibility in WT ESCs (Figure 2D). The ChIP-seq densities of OCT4, SOX2, and NANOG were increased at SOX2-dependent enhancers, with the largest increase displayed by SOX2 (Figure S2C) (Whyte et al., 2013). The stringent consensus SOX2 motif occurred at higher frequency in SOX2-dependent (0.57) OSNs than in SOX2-independent (0.41) OSNs (Figure 2E). Similarly, *de novo* motif discovery identified SOX-OCT and SOX2 motifs whereby occurrence of the latter was significantly higher among SOX2-dependent OSNs (Figure S2D). Thus, SOX2-opened OSNs feature strong SOX2 binding, a highly enriched SOX2 motif, and high accessibility in ESCs.

To assess the functional state of OSNs, we analyzed the local enrichment of a set of histone modifications in ESCs (Figure 2F) (Zhang et al., 2020). Both groups showed high H3K4me1 levels, a signature attributed to distal regulatory elements. In contrast, H3K27Ac, a mark of putative active enhancers, was clearly enriched in SOX2-dependent OSNs (Figure 2G).

We then assessed biological functions of genes adjacent to OSNs using Gene Ontology (GO) analysis. Genes associated with SOX2-independent OSNs were enriched in a wide spectrum of cellular functions and developmental systems ranging from neural to respiratory system development (Figure 2H). In contrast, SOX2-dependent OSN-associated genes displayed strong enrichment in only neural-fate-related GO terms, e.g., “nervous system development” (NSD) (Figure 2H; Table S1). Therefore, we suggest the term SONAEs (SOX2-dependent neural-fate-associated OSN enhancers) for the SOX2-dependent OSNs.

SONAEs were not enriched in motifs of neural TFs such as SOX1, PAX6, NEUROD1, or OCT6 (Figure S2E). Similarly, analysis of the public ChIP-seq data of neural TFs in neural stem cells/neural progenitor cells (NPCs) revealed no preferential binding of these TFs to SONAEs, suggesting that SONAEs are distinct from enhancers occupied by neural TFs and activated in NPCs at later stages of differentiation (Figure S2F) (Thakurela et al., 2016).

### SONAEs are activated immediately following neural induction and essential for neural gene expression

**Figure 3:**
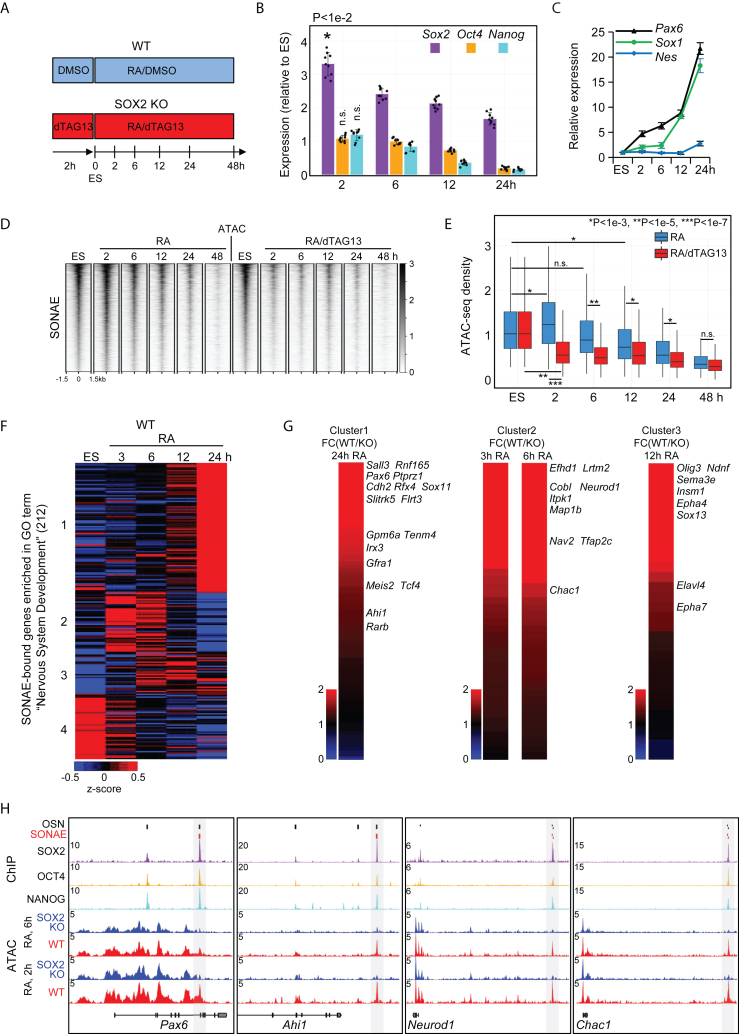
Neural induction immediately up-regulates *Sox2* and activates SONAEs resulting in neural gene expression (A) Schematic representation of RA-induced neural differentiation in WT (RA/DMSO) and SOX2 KO (RA/dTAG13) cells. (B) Bar plot of expression levels in differentiating WT cells (number of independent experiments, n = 3). RT-qPCR was performed using total RNA, and expression levels were calculated relative to ESCs. Data are mean + SEM. p value: paired two-tailed Student’s t test. n.s., not significant. (C) Graph showing expression levels in differentiating WT cells (number of independent experiments, n = 3). Data are mean + SEM. RT-qPCR was performed using total RNA, expression levels were calculated relative to ESCs. (D) Heatmaps of ATAC-seq reads at SONAEs in differentiating WT and SOX2 KO cells. (E) Boxplot of normalized ATAC-seq density at SONAEs upon conditions defined in (A). p values: paired two-tailed Student’s t test. n.s., not significant. Boxplots show median values and first to third interquartile ranges; whiskers: 1.5× the interquartile ranges. (F) K-means-clustered heatmap representation of gene expression as determined by RNA sequencing (RNA-seq) in RA-treated WT cells. (G) Effect of SOX2 KO on the expression of genes from clusters 1, 2, and 3 in (C). The heatmaps show the relative expression of all genes from the corresponding clusters. The values are WT Fragments Per Kilobase of transcript per Million mapped reads (FPKM)/SOX2 KO FPKM. Selected genes are listed on the right according to their positions in the heatmaps. (H) Snapshots of ChIP-seq tracks in ESCs and ATAC-seq tracks in RA-treated WT and SOX2 KO cells. SONAEs are highlighted with gray boxes. See also Figure S3.

To evaluate the role of SONAEs in neurogenesis, we induced neural differentiation with retinoic acid (RA) (Figure 3A). In WT cells, RA induced rapid up-regulation of *Sox2* after 2 h, followed by a reduction between 6 and 24 h (Figure 3B). The levels of *Oct4* and *Nanog* stayed at the ESC level initially but strongly decreased after 12 h. WT cells displayed rapid up-regulation of *Pax6* starting at 2 h, followed by *Sox1* and *Nes*, reflecting the onset of differentiation into neural stem/progenitor cells (Figure 3C) (Thakurela et al., 2016). In accord with the changes in *Sox2* levels (Figure 3B), we found a small but significant transient increase in SONAE accessibility 2 h after RA treatment (Figures 3D and 3E). The accessibility returned to ES levels at 6 h and reached the minimal levels at 48 h (Figure 3E). The increase in accessibility and the retention of open chromatin regions until 24 h were SOX2 dependent since RA-treated SOX2 KO cells exhibited a rapid decrease of accessibility (Figures 3D and 3E). Notably, SOX2-independent OSNs did not display an accessibility increase, confirming the distinctive role of SONAEs (Figures S3A and S3B). Thus, *Sox2* is required for SONAEs’ increased accessibility at the onset of neural differentiation.

However, despite increased *Sox2* expression, SONAEs progressively closed from 12 h after RA treatment. These data suggested that SOX2 may not be sufficient to keep SONAEs open (Figures 3D and 3E). To address this, we performed ChIP-seq for OCT4 and NANOG in RA-treated cells at 6 h. SOX2 depletion reduced OCT4 and NANOG binding to OSNs and SONAEs (Figure S3C). A reduction of OCT4 binding to OSNs by SOX2 KO has also been found previously (Figure S3D) (Friman et al., 2019). Since *Oct4* and *Nanog* are down-regulated upon RA induction, the closure of SONAEs following RA induction is therefore likely due to lack of OCT4 and NANOG (Figure 3B).

To determine whether SONAEs activate the expression of adjacent neural fate genes, we analyzed the transcriptional changes in differentiating cells at 3, 6, 12, and 24 h after RA and evaluated the expression levels of 347 SONAE-adjacent genes associated with the GO term NSD (Table S1). 135 genes that were not expressed were excluded from the analysis. The remaining 212 genes formed four clusters based on their expression in differentiating WT cells (Figure 3F; Table S1). 96 of the 212 genes, including *Pax6*, are associated with 712 DARs characterized by significant accessibility reduction at 12 h of SOX2 depletion (Table S1; Figure S1G). The expression of cluster 1 genes peaked at 24 h, and cluster 2 and 3 genes peaked at 3–6 or 6–12 h, respectively, whereas cluster 4 contained genes down-regulated during differentiation. Thus, SONAE activation highly correlated with expression of NSD genes. To assess whether the expression of said neural fate genes was *Sox2* dependent, we compared the expression levels of genes from each cluster between WT and SOX2 KO cells. Most of the genes were markedly repressed or down-regulated in SOX2 KO cells, whereas only few genes were up-regulated (Figures 3G and S3E; Table S1). Genes critical for early neurogenesis were among the strongest targets of SONAEs (Figure 3G). For instance, genes with the highest fold change (FC) in expression values between WT and SOX2 KO cells in cluster 1 contained a crucial NSD factor, *Pax6*, and the regulators of neural differentiation *Sall3*, *Rnf165*, *Rfx4*, *Sox11*, *Gpm6a*, and *Gfra1* (Zhang et al., 2010; Thakurela et al., 2016). Other neuronal development genes highly dependent on SONAEs included *Efhd1*, *Map1b*, *Neurod1*, *Tfap2c*, and *Chac1* (cluster 2) and *Olig3*, *Sema3e*, *Insm1*, and *Elavl4* (cluster 3) (Figures 3G, 3H, S3E, and S3F; Table S1). Apart from NSD genes, SONAEs are associated with 1,009 genes expressed during RA differentiation (Figure S3G; Table S1). Most (523) were down-regulated, and among the 311 genes up-regulated at 24 h of RA treatment, most (293) were not affected (FC < 2) by SOX2 depletion. Thus, the data suggest that a major role of SONAEs is the activation of early neural control genes (Figures 3G and S3G; Table S1).

As SONAE accessibility gradually decreased from 12 h of RA, we identified enhancers that may regulate NE genes at later stages of differentiation. We detected 5,149 regions whose accessibility significantly increased from 12 to 48 h of RA (Figure S3H). These regions were not bound by SOX2 in ESCs but were strongly bound by SOX2 and PAX6 in NPCs, representing putative NPC enhancers (Figure S3H) (Bergsland et al., 2011; Zhang et al., 2019). 56 out of 93 (60%) SONAE-regulated cluster 1 NSD genes (Figure 3F) were bound by one or more of these regions, suggesting that the latter may take over the control of these NSD genes from SONAEs after their closure (Table S1).

Notably, SOX2-independent OSNs were associated with 646 NSD genes (Figure 2H; Table S1). 237 were also bound by SONAEs. Most of the remaining 409 NSD genes did not undergo significant expression changes during 24 h of RA differentiation (Table S1). However, 193 of the latter genes, including *Sox1*, are associated with putative NPC enhancers, suggesting that their expression may be regulated by these enhancers at later differentiation stages (Figure S3I; Table S1).

### The failure of ME induction in *Smad4* KO cells results in default activation of SONAEs via SOX2

We have recently shown that during BMP4-induced ME differentiation, *Sox2* is repressed by mesodermal TFs, and neural fate genes are not expressed during the differentiation course (Tsaytler et al., 2023). At early stages of WT ESC differentiation, *Sox2* repression is mediated by SMAD4, whereas loss of *Smad4* results in rapid up-regulation of neural fate genes in BMP4-treated cells despite the absence of NE inducers (Tsaytler et al., 2023). Here, we showed that SOX2 ablation in ESCs leads to opening of enhancers bound by ME TFs (Figures S1D–S1F). We set out to investigate whether SONAEs play a role during ME versus NE cell-fate decisions.

**Figure 4:**
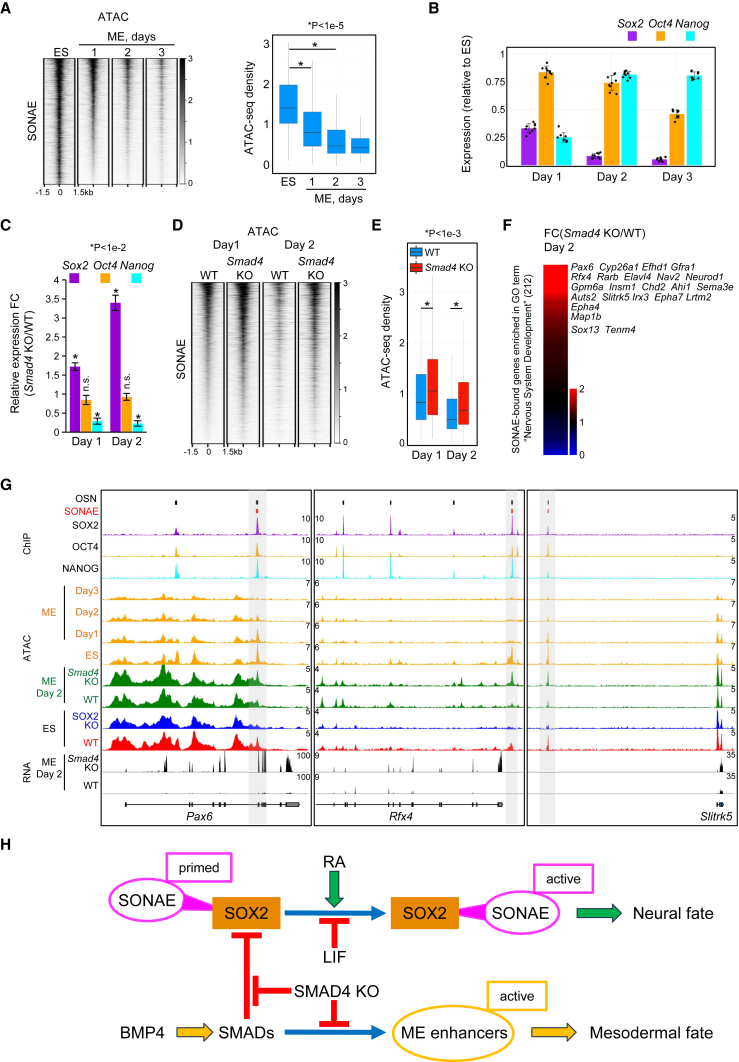
The failure of mesoderm (ME) induction results in SONAE activation by default, followed by neural fate specification (A) Heatmap showing ATAC-seq reads and boxplot of normalized ATAC-seq density at SONAEs during ME differentiation. Each ATAC-seq sample contains merged data from two independent experiments. p value: paired two-tailed Student’s t test. n.s., not significant. Boxplots show median values and first to third interquartile ranges; whiskers: 1.5× the interquartile ranges. (B) Bar plot of RNA expression in ME cells (number of independent experiments, n = 3). Data are mean + SEM. RT-qPCR was performed using total RNA, and expression levels were calculated relative to ESCs. (C) Bar plot of RNA expression in ME-induced *Smad4* KO cells (number of independent experiments, n = 3). RT-qPCR was performed using total RNA, and expression levels were calculated relative to the corresponding WT ME cells. p value: paired two-tailed Student’s t test. n.s., not significant. (D) Heatmap showing ATAC-seq reads at SONAEs in WT and *Smad4* KO ME cells. Each ATAC-seq sample contains merged data from two independent experiments. (E) Boxplot of normalized ATAC-seq density at SONAEs defined in (D). p value: paired two-tailed Student’s t test. Boxplots show median values and first to third interquartile ranges; whiskers: 1.5× the interquartile ranges. (F) Heatmap showing the relative gene expression as determined by RNA-seq. The values are *Smad4* KO FPKM/WT FPKM in ME cells at differentiation day 2. Selected genes are listed on the right according to their positions in the heatmap. (G) Snapshots of ChIP-seq tracks in ESCs, ATAC-seq tracks, and RNA-seq tracks as indicated. SONAEs are highlighted with gray boxes. (H) Schematic view of induced or default neural differentiation via SONAE activation by SOX2. For details, see the main text. See also Figure S4.

SONAEs exhibited rapid closure during ME differentiation, which closely correlated with the down-regulation of *Sox2* but not *Oct4* (Figures 4A and 4B). As ME-differentiated *Smad4* KO cells displayed up-regulation of neural fate genes, we monitored *Sox2* levels in these cells at days 1 and 2 after BMP4 treatment. Whereas the levels of *Oct4* were unaffected and those of *Nanog* were reduced, the levels of *Sox2* were significantly up-regulated in *Smad4* KO cells (Figure 4C). Since repression of *Sox2* in WT ME cells is paralleled by the closure of SONAEs, and in *Smad4* KO ME cells, *Sox2* is up-regulated, we monitored the accessibility of SONAEs in *Smad4* KO ME cells (Figure 4D). As expected, SONAEs were significantly more accessible in ME cells depleted of *Smad4* (Figure 4E), whereas SOX2-independent OSNs were unchanged (Figures S4A and S4B).

We tested whether increased accessibility of SONAEs in *Smad4* KO cells correlated with up-regulation of neural fate genes. A large fraction of genes (119 of 212) identified above as SONAE targets during neural differentiation (Figure 3G) were up-regulated in *Smad4* KO (accessible SONAEs) and repressed in WT (closed SONAEs) ME cells (Figures 4F, 4G, and S4C; Table S1). The strongest up-regulated gene was *Pax6*, while others included *Rfx4*, *Neurod1*, *Gpm6a*, *Insm1*, *Ahi1*, *Irx3*, and *Slitrk5*, and all were associated with one or more SONAEs, which rapidly closed in ME WT cells but remained active in *Smad4* KO ME cells (Figures 4G and S4C).

In sum, loss of *Smad4* results in *Sox2* up-regulation, activation of SONAEs, and induction of NE genes.

## Discussion

In this study, we identified SOX2-dependent OSN enhancers in ESCs and dissected their roles in NE and ME differentiation. We showed that SOX2 directly controls chromatin opening of SOX2-sensitive enhancers (Figures 1D and S1C). In contrast, enhancers showing increased accessibility upon SOX2 loss are controlled via mesodermal factors (Figures S1D–S1F). Although all OSNs bind SOX2, only a fraction of them are sensitive to SOX2 ablation. Here, we identified 2,698 high-confidence SOX2-opened sites, which also showed reduced accessibility upon *Sox2* ablation in public datasets generated using ESCs and EpiLCs, validating our findings (Figures S1H–S1J) (Friman et al., 2019; Maresca et al., 2023; Blassberg et al., 2022).

Our data reveal a role of SOX2-sensitive enhancers beyond pluripotency regulation. We show that only 1,898 out of 8,531 OSNs are affected by SOX2 loss in ESCs (Figure 2B). Depletion of SOX2 diminished the binding of OCT4 and NANOG to SONAEs (Figures S3C and S3D), suggesting that SONAE accessibility also requires OCT4 and/or NANOG in addition to SOX2. We found that SONAEs differ from the rest of OSNs by enhanced SOX2 occupancy, significantly higher accessibility, enriched H3K27Ac marks, and strong association with neural fate genes (Figures 2D–2H and S2C).

The lack of expression of most SONAE-associated neural fate genes in ESCs indicates that SONAEs are not active in the presence of the pluripotency maintenance signal leukemia inhibitory factor (LIF) despite their high accessibility and H3K27Ac levels (Figure 3F; Table S1). This agrees with the findings that enrichment of H3K27Ac and openness alone are not sufficient for enhancer activity (Zhang et al., 2020; Ma et al., 2020). Further, LIF constrains neural activity of *Sox2* even at elevated *Sox2* expression, as cells remain undifferentiated (Zhao et al., 2004). The requirement of *Sox2* for expression of neural fate genes, including *Pax6*, and for NE specification is well established though (Wang et al., 2012; Thomson et al., 2011). We showed that neural induction with RA in the absence of LIF immediately led to transient up-regulation of *Sox2*, selective activation of SONAEs, and consequent up-regulation of SONAE-associated genes critical for neural development, in particular *Pax6*, and including, e.g., *Neurod1*, *Cdh2*, and *Sox11* (Figures 3B–3G). Thus, our results provide insight into the mechanism of how *Sox2* initiates NE specification (Figures 3H and S3F). SONAE activation is transient and gradually decreases from 12 h of RA treatment onwards, concurrent with the opening of a distinct set of putative NPC enhancers, which subsequently take over the control of the neural fate program (Figure S3H). Most of the latter enhancers are co-bound by SOX2 and PAX6, suggesting a feedforward mechanism in early neural specification whereby *Sox2* first activates *Pax6* via SONAEs and then co-regulates neural fate genes together with *Pax6* via NPC enhancers (Figure S3H).

Notably, BMP4-induced ME differentiation promptly repressed *Sox2* and SONAEs (Figures 4A and 4B). ME induction in cells lacking the BMP4 signal transducer *Smad4*, on the other hand, caused up-regulation of *Sox2* and activation of SONAEs, followed by induction of neural fate gene expression (Figures 4C–4G). Thus, *Sox2* is driving cells to the NE fate not just via repression of mesodermal TFs, as suggested previously (Wang et al., 2012; Thomson et al., 2011), but also via activation of SONAEs. Chromatin accessibility is priming active chromatin states and precedes enhancer activation (Ma et al., 2020). The high accessibility of SONAEs in combination with their enrichment for activation-associated histone marks and the rapid activation of SONAEs upon RA induction suggest that SONAEs are primed for rapid selective activation upon neural induction. Furthermore, the onset of the neural fate program induced by LIF withdrawal and high *Sox2* levels in the absence of a neural inducer likely is, at least in part, also mediated by activation of SONAEs (Zhao et al., 2004; Strebinger et al., 2019; Blassberg et al., 2022).

In summary, we characterized the role of *Sox2* in driving NE specification via selective activation of SONAEs, a set of SOX2-dependent neural OSN enhancers. We propose a model whereby the state of SONAEs is an important factor determining lineage decisions in ESCs (Figure 4H). Being highly accessible and primed for activation in ESCs cultured with LIF, SONAEs become active through elevated *Sox2* levels immediately triggered by NE-inducing signals (e.g., RA) promoting neural fate, whereas they rapidly lose accessibility upon *Sox2* repression triggered by ME-inducing signals (e.g., BMP4) promoting the mesodermal fate. Our model also provides an underlying mechanism for the “neural default model” whereby upon withdrawal of pluripotency maintenance (e.g., LIF) and differentiation cues (e.g., *Smad4* KO), ESCs preferentially enter the neural lineage, and this, as we show here, occurs via activation of SONAEs (Figure 4H) (Blassberg et al., 2022; Strebinger, 2019; Stern, 2006).

## Experimental procedures

### Resource availability

#### Lead contact

Information and requests for resources and reagents should be directed to Bernhard G. Herrmann (herrmann@molgen.mpg.de).

#### Materials availability

Please contact Bernhard G. Herrmann for requests and inquiries.

#### Data and code availability

Raw and processed sequencing data were deposited in Gene Expression Omnibus under accession number GEO: GSE240327 (https://www.ncbi.nlm.nih.gov/geo/).

### Experimental model and subject details

F1G4 cells (George et al., 2007) were used to generate the SOX2 KO cell line.

#### *In vitro* differentiation

Neural differentiation was performed as previously described (Bibel et al., 2007).

#### ChIP-seq

ChIP-seq was performed as previously described (Tsaytler et al., 2023).

#### ATAC-seq

ATAC-seq was performed as previously described (Buenrostro et al., 2013).

#### RNA extraction and RNA-seq library preparation

RNA extraction and library preparation were performed as previously described (Koch et al., 2017; Tsaytler et al., 2023).

For detailed information regarding experimental procedures and bioinformatic and statistical analyses, see supplemental experimental procedures.

## Supplemental information

Document S1. Figures S1–S4 and supplemental experimental procedures

Table S1. Enhancer coordinates and gene expression values

Document S2. Article plus supplemental information
