# Supplementary material for: Early neural specification of stem cells is mediated by a set of SOX2-dependent neural-associated enhancers
Source: Stem Cell Reports. 2024 Apr 4;19(5):618–28. doi: 10.1016/j.stemcr.2024.03.003 (PMC11103784; doi:10.1016/j.stemcr.2024.03.003)
Supplement: Document S1. Figures S1–S4 and supplemental experimental procedures [file mmc1.pdf]

Stem Cell Reports, Volume *19*

Supplemental Information

Early neural specification of stem cells is mediated by a set of SOX2-dependent neural-associated enhancers

Pavel Tsaytler, Gaby Blaess, Manuela Scholze-Wittler, Frederic Koch, and Bernhard G. Herrmann

FIGURE S1

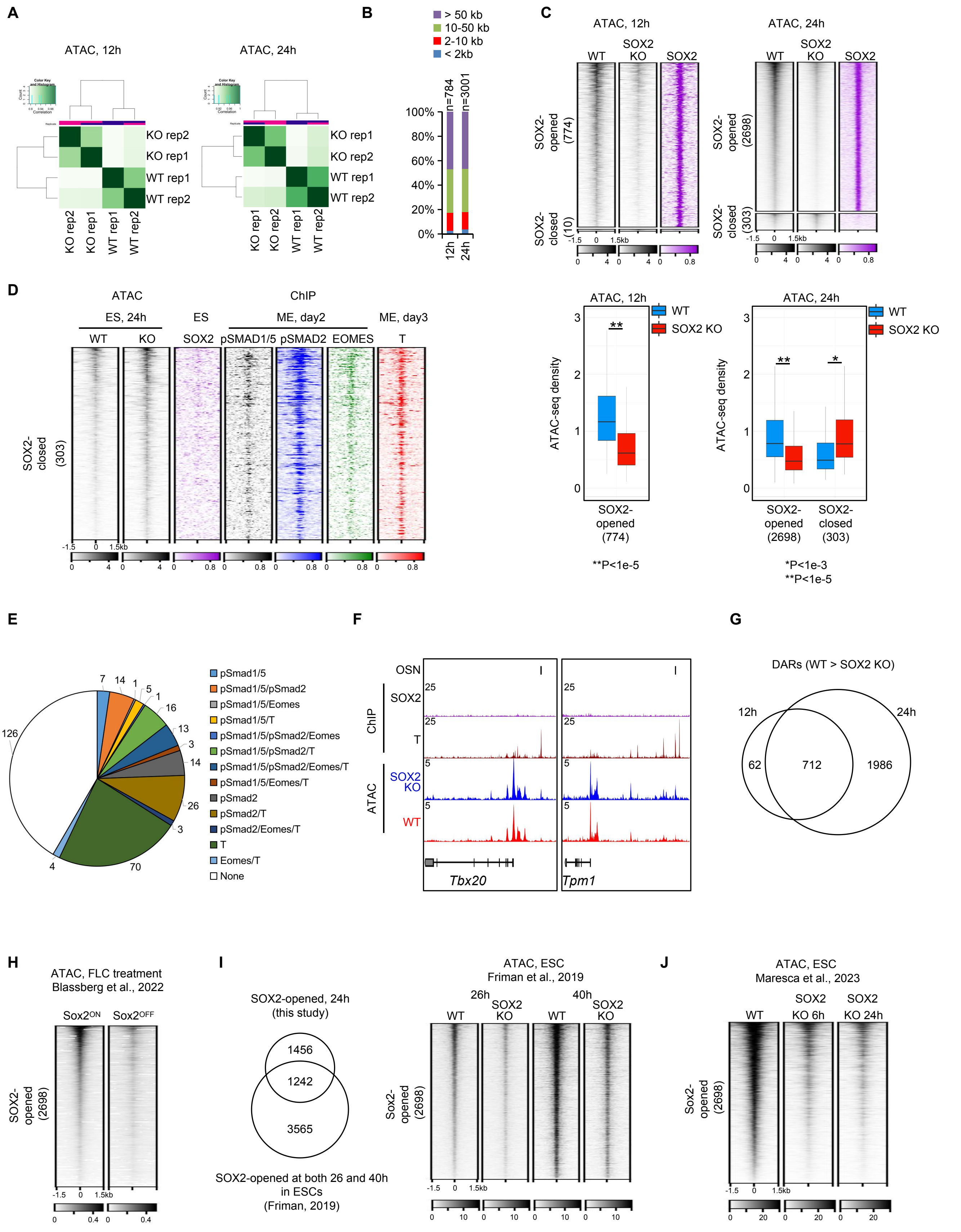
A
ATAC, 12h
ATAC, 24h
KO rep2
KO rep1
WT rep1
WT rep2
B
> 50 kb
10-50 kb
2-10 kb
< 2kb
n=784
n=3001
100%
80%
60%
40%
20%
0%
12h
24h
C
ATAC, 12h
ATAC, 24h
WT
SOX2 KO
SOX2
SOX2-opened (774)
SOX2-closed (10)
SOX2-opened (2698)
SOX2-closed (303)
D
ATAC
ChIP
ES, 24h
ES
ME, day2
ME, day3
WT
KO
SOX2
pSMAD1/5
pSMAD2
EOMES
T
SOX2-closed (303)
ATAC, 12h
ATAC, 24h
WT
SOX2 KO
ATAC-seq density
SOX2-opened (774)
SOX2-opened (2698)
SOX2-closed (303)
**P<1e-5
*P<1e-3
**P<1e-5
E
pSmad1/5
pSmad1/5/pSmad2
pSmad1/5/Eomes
pSmad1/5/T
pSmad1/5/pSmad2/Eomes
pSmad1/5/pSmad2/T
pSmad1/5/pSmad2/Eomes/T
pSmad1/5/Eomes/T
pSmad2
pSmad2/T
pSmad2/Eomes/T
T
Eomes/T
None
F
OSN
ChIP
SOX2
T
ATAC
SOX2 KO
WT
Tbx20
Tpm1
G
DARs (WT > SOX2 KO)
12h
24h
62
712
1986
H
ATAC, FLC treatment
Blassberg et al., 2022
Sox2ON
Sox2OFF
SOX2-opened (2698)
I
SOX2-opened, 24h
(this study)
1456
1242
3565
SOX2-opened at both 26 and 40h
in ESCs
(Friman, 2019)
ATAC, ESC
Friman et al., 2019
26h
40h
WT
SOX2 KO
Sox2-opened (2698)
J
ATAC, ESC
Maresca et al., 2023
WT
SOX2 KO 6h
SOX2 KO 24h
Sox2-opened (2698)

Figure S1. SOX2-dependent differentially accessible regions identified in mouse ESCs (related to Figure 1).

(A) Correlation heatmaps showing clustering of ATAC-seq reads in WT and SOX2 KO replicates using a consensus region set (a total of all regions detected in at least two samples). **(B)** Barplot showing the quantification of distances between DA regions from (B) and nearest gene promoters. **(C)** Clustered heatmaps of ATAC-seq reads in WT and SOX2 KO ESCs and SOX2 ChIP-seq reads in ESCs. Clusters are centered on the DA regions and are ordered top to bottom by ATAC-seq signal intensity in WT ESCs. Clusters contain all detected regions with reduced (top) and increased (bottom) accessibility in SOX2 KO compared to WT cells. Boxplots of the quantification of normalized ATAC-seq density at indicated regions from the heatmaps above. *P<1e-3, **P<1e-5 (paired two-tailed Student's t-test). Boxplots show median values (middle bars), and first to third interquartile ranges (boxes); whiskers indicate 1.5x the interquartile ranges. **(D)** Heatmaps showing ATAC-seq reads in WT and SOX2 KO ESCs, SOX2 ChIP-seq reads in ESCs, and pSMAD1/5, pSMAD2, EOMES, T ChIP-seq reads in ME cells at 303 regions with increased accessibility in SOX2 KO compared to WT cells. **(E)** Pie chart showing regions from (D) occupied by any combination of pSMAD1/5, pSMAD2, EOMES, and T in ME cells. **(F)** Snapshots of SOX2 ChIP-seq in ESCs, T ChIP-seq in ME cells, and ATAC-seq tracks in WT and SOX2 KO cells around the *Tbx20* and *Tpm1* genes. Black and red bars at the top of each panel indicate OSN enhancers. **(G)** Venn diagram showing overlap between regions with reduced accessibility in SOX2 KO compared to WT cells detected after 12 and 24 hours of SOX2 ablation. **(H)** Heatmap showing ATAC-seq reads in FGF/LGK974/CHIR99021(FLC)-treated Sox2ON and Sox2OFF cells generated by [Blassberg, 2022] around the 24 h SOX2-opened regions (C,G). **(I)** Venn diagram showing overlap between the 24 h SOX2-opened regions identified here and SOX2-opened regions identified in [Friman, 2019]. The modest overlap presumably is due to incomplete Sox2 protein ablation at 26 h after dox induction, since at 40 h after dox treatment nearly all 2698 SOX2-opened sites showed markedly decreased chromatin accessibility on the heatmap to the right. Heatmap showing ATAC-seq reads in 26 and 40 h WT and *Sox2* KO cells generated by [Friman, 2019] around the 24 h Sox2-opened regions. **(J)** Heatmap showing ATAC-seq reads in WT and 6 and 24 h SOX2 KO ESCs generated by [Maresca, 2023] around the 24 h SOX2-opened regions.

FIGURE S2

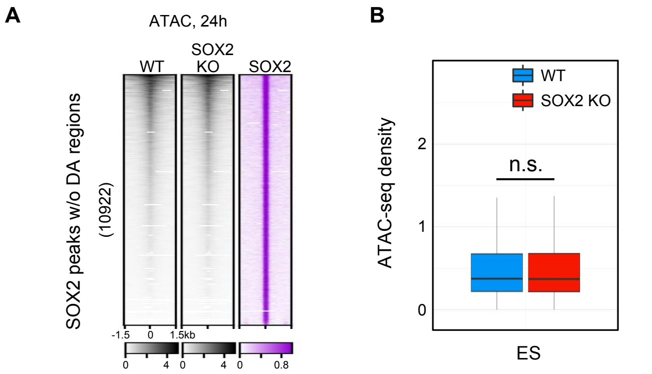

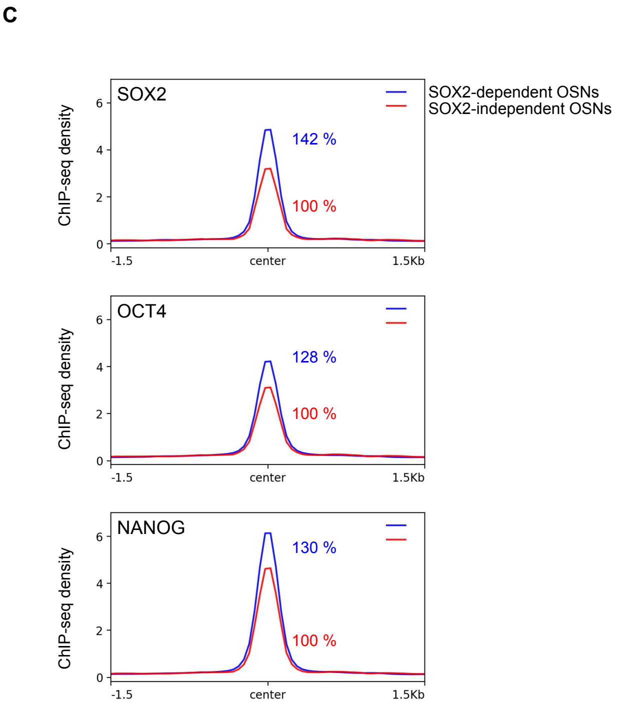

D

SOX2-dependent OSNs (1898)

Motif	best match	e-value	sites contributing to motif (% of total sites)
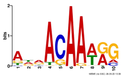	SOX2	1.1e-414	1897 (99.9%)
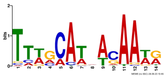	SOX-OCT	4.2e-633	1453 (76.6%)

SOX2-independent OSNs (6633)

Motif	best match	e-value	sites contributing to motif (% of total sites)
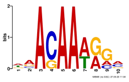	SOX2	7.1e-174	4024 (60.6%)
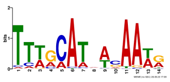	SOX-OCT	5.8e-460	4017 (60.5%)

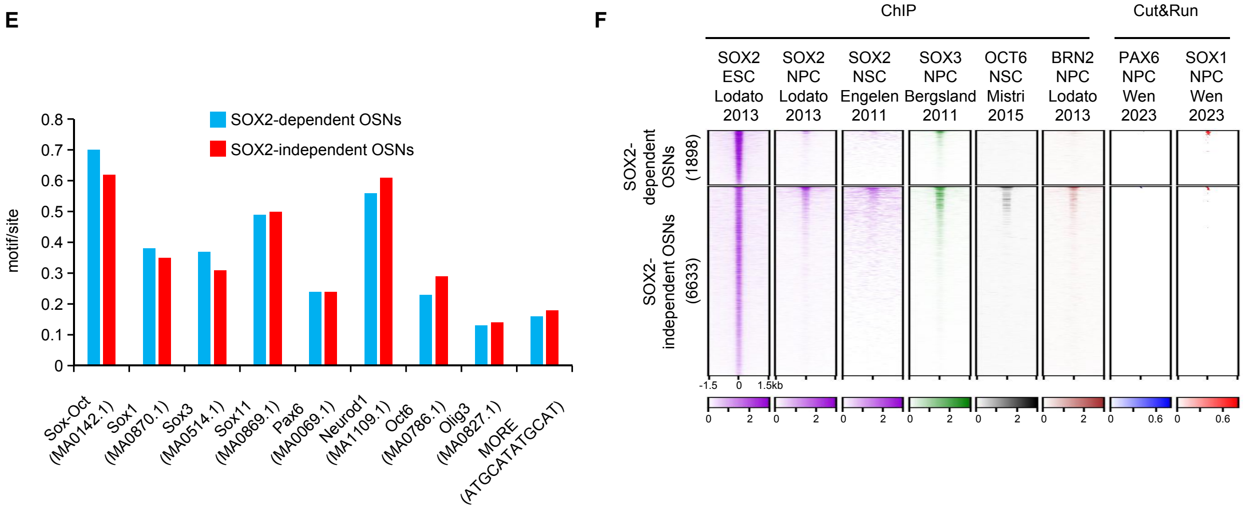

Figure S2. Chromatin accessibility and TF binding at OSN enhancers (related to Figure 2).

(A) Heatmaps of ATAC-seq reads in WT and SOX2 KO ESCs and SOX2 ChIP-seq reads in ESCs around 10922 SOX2 peaks that display no change in accessibility levels upon SOX2 ablation (Fig. 1G). **(B)** Boxplot of the quantification of normalized ATAC-seq density at 10922 SOX2 peaks from (A). n.s., not significant (paired two-tailed Student's t-test). Boxplots show median values (middle bars), and first to third interquartile ranges (boxes); whiskers indicate 1.5x the interquartile ranges. **(C)** Average SOX2, OCT4, and NANOG ChIP-seq density profiles in WT ESCs in SONAEs (blue) and the rest of OSN enhancers (red). Percentages indicate the relative increase of ChIP-seq read densities in SONAEs compared to the rest of OSN enhancers. **(D)** Motifs identified among SOX2-dependent and SOX2-independent OSNs using *de novo* MEME motif discovery tool. **(E)** Barplot showing the occurrence of indicated motifs in SOX2-dependent and SOX2-independent OSNs. **(F)** Heatmaps of indicated public ChIP-seq and Cut&Run reads at SOX2-dependent and SOX2-independent OSNs.

FIGURE S3

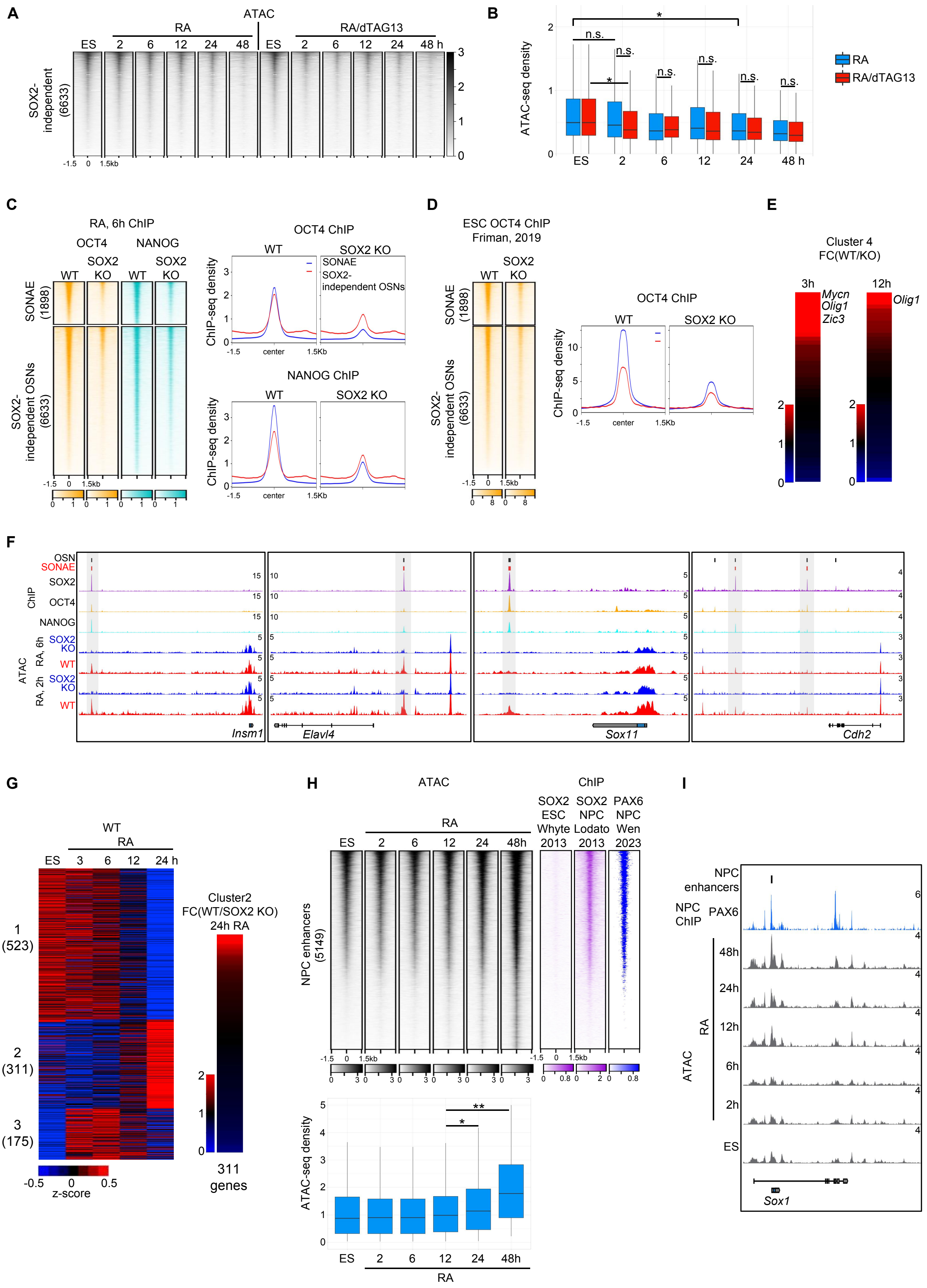
A
ATAC
RA
RA/dTAG13
ES 2 6 12 24 48
ES 2 6 12 24 48 h
SOX2-independent (6633)
-1.5 0 1.5kb
B
ATAC-seq density
n.s.
*
RA
RA/dTAG13
ES 2 6 12 24 48 h
C
RA, 6h ChIP
OCT4
NANOG
SOX2 KO
WT
SONAE (1898)
SOX2-independent OSNs (6633)
OCT4 ChIP
NANOG ChIP
ChIP-seq density
SONAE
SOX2-independent OSNs
center
1.5Kb
D
ESC OCT4 ChIP Friman, 2019
E
Cluster 4 FC(WT/KO)
3h
12h
Mycn
Olig1
Zic3
F
OSN
SONAE
SOX2
OCT4
NANOG
ChIP
ATAC
RA, 6h
RA, 2h
SOX2 KO
WT
Insm1
Elavl4
Sox11
Cdh2
G
WT
RA
ES 3 6 12 24 h
1 (523)
2 (311)
3 (175)
z-score
-0.5 0 0.5
Cluster2 FC(WT/SOX2 KO) 24h RA
311 genes
H
ATAC
ChIP
SOX2 ESC Whyte 2013
SOX2 NPC Lodato 2013
PAX6 NPC Wen 2023
NPC enhancers (5149)
ES 2 6 12 24 48h
ATAC-seq density
**
I
NPC enhancers
NPC ChIP
PAX6
48h
24h
12h
6h
2h
ES
Sox1

Figure S3. Chromatin accessibility at SOX2-independent OSN enhancers (related to Figure 3).

(A) Heatmaps of ATAC-seq reads at SOX2-independent OSN enhancers in WT (RA) and SOX2 KO (RA/dTAG) treated cells. **(B)** Boxplot of the quantification of normalized ATAC-seq density at SOX2-independent OSN enhancers upon conditions defined in (A).*P<1e-3, **P<1e-5 (paired two-tailed Student's t-test). n.s., not significant. Boxplots show median values (middle bars), and first to third interquartile ranges (boxes); whiskers indicate 1.5x the interquartile ranges. **(C)** Heatmaps and average density profiles for OCT4 and NANOG ChIP-seq reads in WT and SOX2 KO RA differentiated cells at 6 h SOX2 ablation results in reduced reqruitment of OCT4 and NANOG to SOX2-independent OSNs and, to a larger extent, to SONAE. **(D)** Heatmaps and average density profiles for OCT4 ChIP in WT and SOX2 KO ESCs from [Friman, 2019]. **(E)** The effect of SOX2 KO on the expression of genes from cluster 4 (Fig. 3F). The heatmaps show the relative expression of all genes from the cluster. The values are WT FPKM/SOX2 KO FPKM at 3 and 12 h after RA treatment. Selected genes are listed on the right according to their positions in the heatmaps. **(F)** Snapshots of SOX2, OCT4, and NANOG ChIP-seq tracks in ESCs [Whyte, 2013] and ATAC-seq tracks in RA-treated WT and SOX2 KO cells around the *Insm1*, *Elavl4*, *Sox11*, and *Cdh2* genes. Black and red bars at the top of each panel indicate total OSN enhancers and SONAEs, respectively. SONAEs are also highlighted with gray boxes. **(G)** (left) K-means clustered heatmap representation of expression of 1009 genes (FPKM > 1) associated with SONAE (excluding NSD genes), as determined by RNA-seq in RA-treated wild-type (WT) cells. (right) The effect of SOX2 KO on the expression of 311 genes from cluster 2. The heatmap show the relative expression of all genes from the cluster. The values are WT FPKM/SOX2 KO FPKM at 24 h after RA treatment. Expression of most of the genes is not affected by SOX2 KO, in contrast to NSD genes (Fig. 3G). **(H)** Heatmaps showing ATAC-seq reads in RA-induced neural differentiated WT cells (left) at putative NPC enhancers identified based on increased accessibility in 24 or 48 h of RA compared to WT. Heatmaps of ChIP-seq reads for SOX2 in ESCs [Whyte, 2013], SOX2 in NPCs [Lodato, 2013], and PAX6 in NPCs [Wen, 2023] at putative NPC enhancers (right). Boxplot of the quantification of normalized ATAC-seq density at NPC enhancers.*P<1e-3, **P<1e-5 (paired two-tailed Student's t-test). Boxplots show median values (middle bars), and first to third interquartile ranges (boxes); whiskers indicate 1.5x the interquartile ranges. (bottom). **(I)** Snapshot of NPC PAX6 ChIP-seq and RA-induced differentiation ATAC-seq tracks at the *Sox1* gene locus. Black bar indicates a putative NPC enhancer.

FIGURE S4

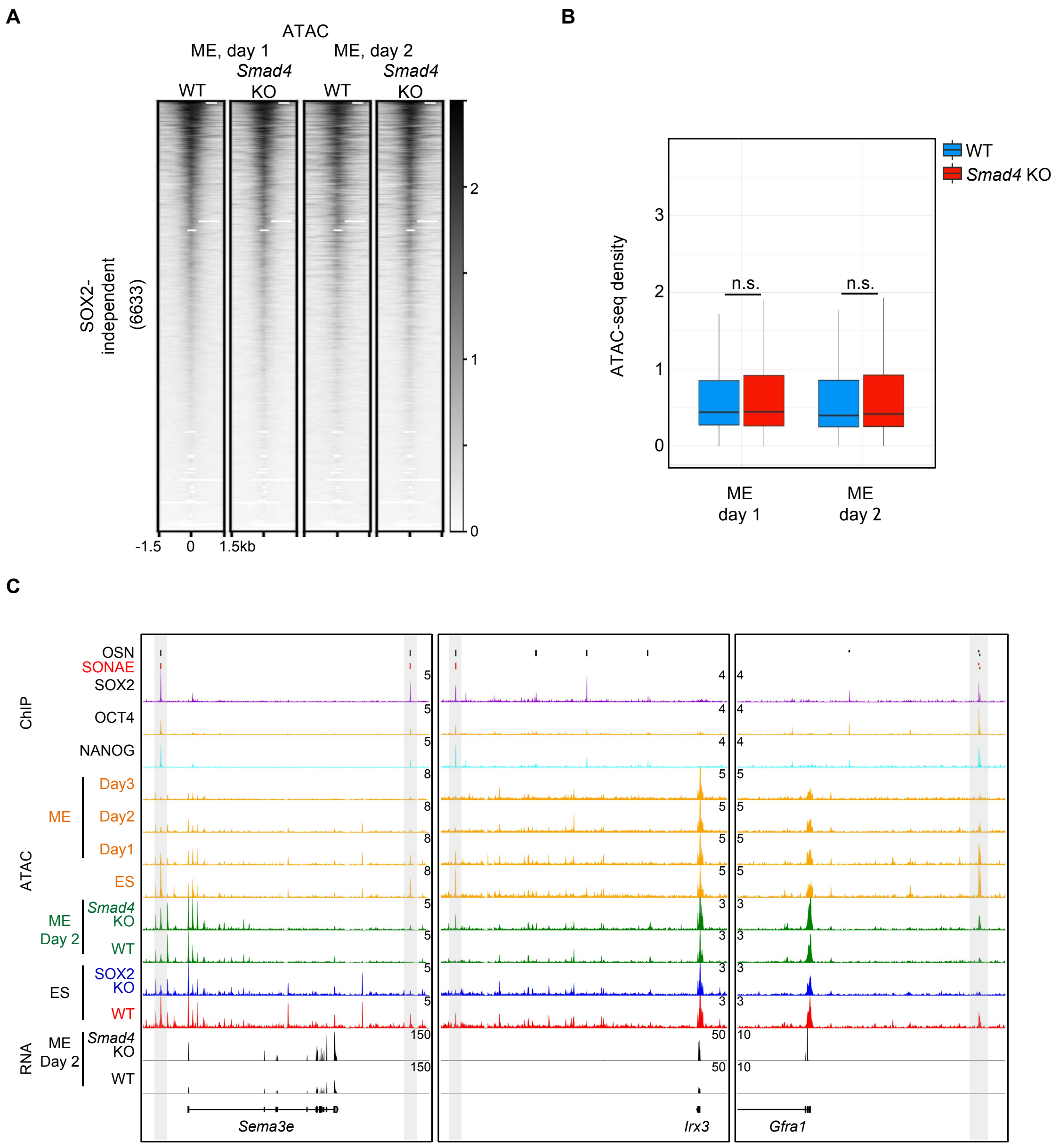

A
ATAC
ME, day 1
ME, day 2
WT
Smad4 KO
WT
Smad4 KO
SOX2-independent (6633)
-1.5
0
1.5kb
2
1
0
B
WT
Smad4 KO
ATAC-seq density
n.s.
n.s.
3
2
1
0
ME day 1
ME day 2
C
OSN
SONAE
SOX2
OCT4
NANOG
ChIP
ME
Day3
Day2
Day1
ES
ATAC
ME Day 2
Smad4 KO
WT
ES
SOX2 KO
WT
RNA
ME Day 2
Smad4 KO
WT
Sema3e
Irx3
Gfra1

Figure S4. SOX2-independent OSN enhancers are not affected by mesoderm induction failure (related to Figure 4).

(A) Heatmap showing ATAC-seq reads at OSN enhancers excluding SONAEs in WT and *Smad4* KO ME cells. **(B)** Boxplot of the quantification of normalized ATAC-seq density at SONAEs upon conditions defined in (A). n.s., not significant (paired two-tailed Student's t-test). Boxplots show median values (middle bars), and first to third interquartile ranges (boxes); whiskers indicate 1.5x the interquartile ranges. **(C)** Snapshots of SOX2, OCT4, and NANOG ChIP-seq tracks in ESCs, ATAC-seq tracks in WT and *Smad4* KO ME cells and in WT and SOX2 KO ESCs, and RNA-seq tracks in WT and *Smad4* KO ME cells around the *Sema3e*, *Irx3*, and *Gfra1* genes. Black and red bars at the top of each panel indicate OSN enhancers and SONAEs, respectively. SONAEs are highlighted with gray boxes.

Supplemental experimental procedures

Culture of mouse embryonic stem cells (mESCs)

All mESC lines were maintained on plates coated with 0.1% gelatin (Sigma-Aldrich; G1393) on a layer of mitotically inactive primary mouse embryo fibroblasts (feeder cells) in ESC growth medium: knockout Dulbecco's Modified Eagle's medium (DMEM) (ThermoFisher; 10829018) supplemented with 2mM L-glutamine (Lonza; BE17-605E), 15% (v/v) heat-inactivated FCS, 1% (v/v) non-essential amino acids (ThermoFisher; 11140050), 0.1 mM β-mercaptoethanol (Sigma-Aldrich; M3148), 1% (v/v) nucleosides (Sigma-Aldrich; ES-008-D), 1% (v/v) penicillin and streptomycin (Lonza; DE17-603E), and 1000 U/ml LIF (Chemicon; ESG1107) at 37°C and 7.5% CO_2 .

Generation of SOX2 KO mESC line

To generate SOX2-FKBP12 cells, a plasmid containing a 3xG4S linker, the FKBP12 binding domain, pA signal and a PGK-Neomycin-SV40pA selection cassette was generated and flanked by 2kb and 3.5kb homologous relative to the *Sox2* translation stop, respectively. For transfection, 50µg of the plasmid was linearized and electroporated into $10x10^6$ mouse F1G4 cells. Cells were selected using 350µg/ml G418 and single colonies were picked into 96 well plates and expanded. At average confluence, the plates were split, frozen and one fraction was used further to expand and purify DNA for genotyping via PCR on the 5' (F:GATGGGGCAGGTTGGTGGAG; R:GGTGTAGTGCACCACGCAGG) and 3' (F: GAGGCGGAAAGAACCAGCTGG; R:CCACTGCAAGAATGTGTCGC) regions. Heterozygous clones were chosen and the expression of the SOX2-FKBP12 fusion protein was verified via Western blot.

The WT allele was deleted using CRISPR/Cas9 with the double nickase approach using the px335A_hCas9_D10A_G2P plasmid (a gift from Boris Greber, Max Planck Institute for Molecular Biomedicine, Münster, Germany). The primers used for cloning of the guide RNA's were: sox2_sg1_top: CACCGAGGAGCCGGCGCTCGCTGAT; sox2_sg1_bot: AAACATCAGCGAGCGCCGGCTCCTC; sox2_sg2_top: CACCGCCGAGCCCAGCCTCGCCAGA; sox2_sg2_bot: AAACTCTGGCGAGGCTGGGCTCGGC; sox2_sg3_top: CACCGCACAAAGAAATGAAAGACTC; sox2_sg3_bot: AAACGAGTCTTTCATTTCTTTGTGC; sox2_sg4_top: CACCGCATGCTTATACATAACTTCT; sox2_sg4_bot: AAACAGAAGTTATGTATAAGCATGC. Co-tranfections of the resulting 4 plasmids were performed using Lipofectamine (Invitrogen 11668027) as previously described (Schifferl et al., 2021). Genotyping was performed via PCR (F: GATGGGGCAGGTTGGTGGAG; R: CATCGCGCTTATTCACGCTGC) and sequencing of the resulting PCR product. The resulting clones were again validated using Western blotting.

In Vitro Differentiation

Neural differentiation of mESCs was performed as previously described [Bibel, 2007] with minor modifications. Briefly, SOX2-FKBP12 mESCs were deprived of feeder cells by dissociating in trypsin and passaging for four consecutive 25 minute periods, followed by a 24 hour period. Cells were then dissociated, and 40,000 cell/ml cell suspension was plated onto tissue culture plates in 5 µl droplets using electronic multi-channel pipettes. Plates were then inverted and incubated for 24 hours. Drops containing mES cell aggregates (embryoid bodies) were then pooled and washed with ESC growth medium. mESC aggregates were then resuspended in ESC growth medium without LIF and incubated in 6-well cell culture plates for 48 hours. To induce SOX2 protein degradation, dTAG13 (Tocris, 6605) was added to the medium at a final concentration of 250 nM for 2 hours prior to the induction of differentiation. The corresponding amount of DMSO was added to the control (WT) cells. To start differentiation, the medium was replaced with ESC growth medium without LIF containing 5 µM retinoic acid (Sigma-Aldrich; R2625) and 250 nM dTAG13 (SOX2 KO) or DMSO (WT).

Antibodies

The antibodies used for western blotting: SOX2 (R&D Systems, AF2018), OCT4 (Santa Cruz, sc-8628), NANOG (Abcam, ab80892), GAPDH (Cell Signaling, 5174). The antibodies used for ChIP-seq: NANOG (Bethyl Laboratories, A300-397A), OCT4 (Santa Cruz, sc-8628).

Analysis of Protein Expression Levels by Western Blotting

Whole cell extracts were prepared from mESCs using 1X Novex NuPAGE LDS sample buffer (ThermoFisher; NP0007) supplemented with 100 mM DTT (Sigma-Aldrich; 43815). DNA was digested using Benzonase Nuclease (Millipore; E1014). Samples were heated at 95°C for 5 minutes, run on 4-12% NuPAGE Bis-Tris Protein Gel, transferred to nitrocellulose membrane, and subjected to western blotting using primary antibodies at 1:1000 dilution and the appropriate HRP-conjugated secondary antibodies at 1:5000 dilution. Detection was performed using the Amersham ESL reagents (GE Healthcare) and scanned using a Fusion SL chemiluminescent detection system (Vilber). At least two biological replicates were performed for each experiment. The Fusion software was used for quantification. GAPDH protein levels were used for normalization.

qRT-PCR analysis
For RNA extraction, cells were collected at the indicated times and processed with the RNeasy Micro kit (Qiagen). For each sample, 500 ng of total RNA was used for cDNA synthesis with QuantiTect Reverse Transcription Kit (Qiagen). Quantitative PCR was performed on a StepOnePlus Real-Time PCR System (Applied Biosystems) using Pmm2 as internal control gene for calculating relative expressions. Sequences of primers used for qRT-PCR: Sox2 (F:AGCGCATGGACAGCTACG; R:CATCGGTTGCATCTGTGC), Oct4 (F:TCAGCTTGGGCTAGAGAAGG; R:TGGGAAAGGTGTCCCTGTAG), Nanog (F:AAACCAAAGGATGAAGTGCAAG; R:GGATACTCCACTGGTGCTGAG), Pax6 (F:CCTCGCCTCCAGCCTCAG; R:GGTTAAAGTCTTCTGCCAAGAGC), Sox1 (F:CATGCACCGCTACGACATGG; R:TAAGGGATGCCGCCGTAGC), Nes (F:GAGAGGCGCTGGAACAGAG; R:TTCCACAGCCAGCTGGAAC), Pmm2 (F:ACTGGGAAATGATGAGAATGGC; R:CCCCCAGATGCCCTTGAATA). At least three independent experiments were performed for each analysis. Data shown on barplots and graphs are mean + s.e.m. For statistical analysis, paired two-tailed Student's *t*-test was used.

RNA extraction and RNA-seq Library Preparation
RNA extraction and library preparation was performed as previously described [Koch, 2017]. Briefly, total RNA was extracted from 40,000 mESCs or differentiated cells using Trizol reagent (ThermoFisher; 15596026) following the manufacturer's instructions and purified using the RNeasy Micro kit (Qiagen). Residual genomic DNA was digested on a column. The RNA was quantified using Qubit RNA HS assay (Life Technologies), and the integrity of the RNA was assessed using Bioanalyzer RNA pico chips (Agilent).
Strand-specific RNA-seq libraries were generated from 100 ng of total RNA using the ScriptSeq v2 (Epicentre) low input library preparation kit according to manufacturer's instructions. The library was amplified using 15 PCR cycles. RNA-seq libraries were quantified using the Qubit HS DNA assay (Life Technologies) and the size distribution was assessed using the DNA HS Bioanalyzer chips (Agilent). Libraries were pooled and paired-end sequenced on the NovaSeq 6000 (Illumina) with 100 bp read lengths or NextSeq2000 (Illumina) with 50 bp read lengths.

ATAC-seq
ATAC-seq was performed as previously described [Buenrostro, 2013]. The cells were trypsinized, and the trypsin was inactivated by washing with cold PBS containing 2% BSA. For each sample, 50,000 cells were collected, washed with 1 ml cold PBS and pelleted by centrifugation. The cells were lysed in 50 µl of cold lysis buffer (10 mM Tris pH 7.4, 10 mM NaCl, 3 mM $MgCl_2$, 0.1% IGEPAL CA-630) and immediately centrifuged at 500g, 4°C for 10 min. The pellet was resuspended in the transposition reaction mix (25 µl 2x TD buffer, 2.5 µl Tn5 transposase, 22.5 µl H_2O) and incubated at 37°C for 30 minutes. After incubation, the reaction was stopped with the addition of PB buffer (Qiagen) and the tagmented DNA was purified using the MinElute kit (Qiagen). The DNA was combined with the ATAC index PCR primers and 2x Kapa HiFi Hotstart Ready Mix and pre-amplified (98°C 30 seconds, 5x [98°C 10 seconds, 63°C 30 seconds, 72°C 1 minute] in a 50 µl volume. To determine the remaining cycles to avoid potential over-amplification, 5 µl of the pre-amplification mix was combined with the primers, 1x Evagreen SYBR green (Jena Biosciences) and 2x Kapa HiFi Hotstart Ready Mix in a 15 µl total volume and run for 30 cycles on a StepOne Plus. The remaining 45 µl of pre-amplified samples were amplified for a further 6-7 cycles and the libraries were purified using MinElute column (Qiagen). The libraries were quantified using the Qubit DNA HS assay

and the library sizes were validated using DNA HS Bioanalyzer chips. Samples were pooled and paired-end sequencing was performed on the NovaSeq 6000 with 100 bp read lengths.

Chromatin Immunoprecipitation (ChIP)

Crosslinking was performed directly on differentiating cells in differentiation medium with the addition of 1/10th volume of crosslinking solution (11% formaldehyde, 50 mM Hepes pH 7.8, 100 mM NaCl, 1 mM EDTA, 0.5 mM EGTA) for 10 minutes at room temperature, while shaking. The crosslinking reaction was quenched with the addition of 1/10th volume of 2.5 M glycine and 5 minutes incubation. Cells were washed twice with cold PBS, scraped in cold PBS containing 0.05% Triton X-100, and pelleted in aliquots of $5x10^7$. For sonication, complete protease inhibitors without EDTA (Roche) at 1x final concentration was added to all lysis buffers (LB) prior to use. Each pellet was resuspended in 2.5 ml LB1 (50 mM Hepes pH 7.5, 140 mM NaCl, 1 mM EDTA, 10% glycerol, 0.75% NP-40, 0.25% Triton X-100) and rotated at 4°C for 20 minutes. The cell suspension was homogenized using a douncer. The chromatin was pelleted by centrifugation at 1400g and 4°C for 5 minutes and resuspended in 2.5 ml LB2 (10 mM Tris pH 8, 200 mM NaCl, 1 mM EDTA, 0.5 mM EGTA). After 10 minutes rotation at 4°C, the centrifugation step was repeated and each pellet was resuspended in 1.5 ml LB3 (10 mM Tris pH 8, 1 mM EDTA, 0.5 mM EGTA, 100 mM NaCl, 0.1% Na-deoxycholate, 0.5% N-lauroylsarcosine), transferred to 15 ml Falcon tubes and sonicated using a W-450D Digital Sonifier (Branson) sonicator for 14 cycles of 10 seconds on/50 seconds off in a 4°C cold room with tubes chilled in ice water. After sonication, 150 µl of Triton X-100 was added per tube, transferred to two 1.5 ml Eppendorf tubes and debris was pelleted by centrifugation at 20,000g and 4°C for 10 minutes. The solubilized chromatin was then pooled and mixed thoroughly. The chromatin was distributed into 1.5 ml aliquots, snap frozen and stored at -80°C until use.
100 µl protein G-coated Dynal beads (Life Technologies) were washed 3x with 1 ml of blocking buffer (PBS, 0.5% BSA), resuspended in 500 µl of blocking solution containing 5 µg ChIP antibody and rotated overnight at 4°C. Beads were then washed 3x with 1 ml of blocking buffer and resuspended in 100 µl blocking buffer. Chromatin equivalent to $5x10^7$ cells was added and rotated overnight at 4°C. The following day, 6 washing steps (9 for T) with 1 ml RIPA buffer (50 mM Hepes pH 7.6, 500 mM LiCl, 1 mM EDTA, 1% NP-40, 0.7% Na-Deoxycholate) and one washing step with 1 ml of TEN (10 mM Tris pH 8, 1 mM EDTA, 50 mM NaCl) were performed. The elution was performed in two subsequent steps using 100 µl of 1x elution buffer (50 mM Tris pH 8, 10 mM EDTA, 1% SDS) and incubation at 65°C while shaking for 10 minutes each. The eluates were combined and incubated for 13-15 hours at 65°C. 200 µl of TE were added and the ChIP DNA was purified as described for the input above. ChIP DNA was quantified using the Qubit (Life Technologies) DNA HS assay.

ChIP-seq Library Preparation

The ChIP-seq libraries were generated using the TruSeq ChIP library kit (Illumina) with the following modifications. After adapter ligation, 0.95x of AMPure XP beads (Beckman Coulter) were used for a single purification and the DNA was eluted using 14 µl of resuspension buffer (RSB, Illumina). After the addition of 1 µl primer mix (25 mM each, Primer 1: 5'-AATGATACGGCGACCACCGA*G-3'; Primer2: 5'- CAAGCAGAAGACGGCATACGA*G-3') and 15 µl 2x Kapa HiFi HotStart Ready Mix (Kapa Biosystems), amplification was performed for 45 seconds at 98°C, 5 cycles of [15 seconds at 98°C, 30 seconds at 63°C and 30 seconds at 72°C] and a final 1 minute incubation at 72°C. The PCR products were purified using 0.95x of beads and eluted using 21 µl of RSB. Ligation products were then separated using a 1.5% agarose gel. Post-run staining was performed using SYBR Gold (Life Technologies) under agitation for 30 minutes. Gel slices corresponding to ~250-400 bp fragment size were cut out using a Dark Reader (Clare Chemical Research) transilluminator. The gel extraction was performed using 5 gel volumes of QG buffer (Qiagen) with the addition of one gel volume of isopropanol. The MinElute (Qiagen) columns were washed once with QG buffer and twice with PE buffer, air-dried for at least 10 minutes and eluted using 21 µl of EB buffer. 19 µl of the eluate was used in the final amplification, with the addition of 1 µl primer mix and 20 µl 2x Kapa HiFi Hotstart premix. The same protocol as for the pre-amplification was used, with the exception of using 13 amplification cycles. The libraries were quantified using the Qubit DNA HS assay and the library size was validated using DNA HS Bioanalyzer chips. Sequencing was performed on the NextSeq 2000 using 2x 50 bp read length.

QUANTIFICATION AND STATISTICAL ANALYSIS

Genome Assembly

All datasets were mapped to the mouse mm10 reference genome containing chromosomes 1-19, X, Y, and M.

Analysis of RNA-seq data

RNA-seq reads were subjected to quality control using FastQC (https://www.bioinformatics.babraham.ac.uk/projects/fastqc/) and trimmed using cutadapt (version 2.9) (https://doi.org/10.14806/ej.17.1.200) to remove adapter sequences. Alignment was performed using Hisat2 [Kim, 2019] (version 2.1.0) with the following arguments: *--no-mixed --no-discordant*. The index files for Hisat2 were built using mm10 reference genome containing chromosomes 1-19, X, Y and M, and splice sites and exon data from refSeq annotations. The resulting sam format files were then converted to bam format, sorted and indexed using samtools [Li, 2009] (version 1.9). FPKM values were calculated using Cuffdiff [Trapnell, 2010] (version 2.2.1) with the following arguments: *-u -no-effective-length-correction -b*.

Statistical analysis of differential gene expression was performed using Rsubread featureCounts [Liao, 2014] and DESeq2 [Love, 2014]. First, tables containing unprocessed read counts for technical WT and SOX2 KO RNA-seq replicate experiments were generated using featureCounts with the following arguments: isPairedEnd = TRUE, requireBothEndsMapped = TRUE. For each neural differentiation time point, pairwise WT-SOX2 KO statistical analysis was performed using DESeq2 with default settings. The cutoffs used for statistical significance were as follows: adjusted p value (padj) < 0.005, absolute fold change > 1.25.

For clustering of genes associated with the GO-term ‘Nervous System Development’, the k-means clustering into 4 groups was performed using Cluster3.0 [De Hoon, 2004], and visualized using Java TreeView [Saldanha, 2004].

For visualization the data were normalized and converted to bigWig (bw) format using bamCoverage tool from deepTools [Ramirez, 2016] (version 3.1.3) with the following arguments: *--normalizeUsing BPM -bs 5 --ignoreDuplicates*. Genome browser snapshots containing RNA-seq data were generated using pyGenomeTracks [Ramirez, 2018] (version 3.1). The expression data were visualized in heatmaps using heatmap.2 function of gplots package (version 3.0.1) (https://CRAN.R-project.org/package=gplots) in R.

Analysis of ATAC-seq data

Two independent ATAC-seq experiments for each WT and SOX2 KO time point were performed to identify SOX2-dependent DARs. ATAC-seq reads were trimmed to 40 bp length and adapter sequences were removed using cutadapt. The trimmed reads were aligned to the reference genome using bowtie [Langmead, 2009] (version 1.1.2) with the following arguments: *-y -v 2 --best --strata -m 3 -k 1 -S -X 2000 --allow-contain*. Sam files containing mapped paired-end reads were converted to bam files, sorted and indexed using samtools. Reads mapped to chr M and Y or known ATAC artifact regions (ENCODE) were removed using samtools. Possible PCR duplicates were removed using Picard (version 1.103, https://broadinstitute.github.io/picard).

For visualization, the reads from independent experiment replicates were combined, and the data were normalized and converted to bw format using bamCoverage tool from deepTools with the following arguments: *--normalizeUsing RPCG --effectiveGenomeSize 2652783500 --extendReads 200 --smoothLength 9 -bs 3 -ignore chrY chrM*. All accessible regions were detected as narrowPeaks using MACS2 [Zhang, 2009] with q value cutoffs of 5e-2.

To identify regions that are differentially accessible between WT and SOX2 KO cells and to perform correlation analysis between WT and SOX2 KO replicates, we used DiffBind package (version 2.12.0, https://bioconductor.org/packages/release/bioc/html/DiffBind.html) in R. As input, for each replicate of WT and SOX2 KO ATAC-seq data, bam file containing filtered mapped reads and list of genomic coordinates of accessible regions were used. DiffBind was run with default parameters, except consensus peak list was set to include peaks detected in at least two of the four samples.

Detected ATAC-seq regions were considered to overlap with ChIP-seq peaks if the summit of a ChIP-seq peak was located within an ATAC-seq region. ATAC-seq and ChIP-seq heatmaps,

with clustering based on the differential accessibility were generated using computeMatrix and plotHeatmap tools from deepTools. ChIP-seq density profiles were generated using plotProfile tool from deepTools. Normalized ATAC-seq bw files and coordinates of ATAC-seq region centers or SOX2 ChIP-seq peak summits were used as input. Boxplots indicating distributions of ATAC-seq or ChIP-seq densities within heatmap clusters were generated using ggplot2 (version 3.1.1, https://ggplot2.tidyverse.org/) package in R. The values were obtained from the output file of *--outFileNameMatrix* argument of the computeMatrix tool run with additional argument *-bs 1500.* For statistical analysis, paired two-tailed Student's *t*-test was used. Boxplots show median values (middle bars), and first to third interquartile ranges (boxes); whiskers indicate 1.5x the interquartile ranges.

Analysis of ChIP-seq data

The reads were aligned to the reference genome using bowtie2 (version 2.2.4) with the following arguments: *-3 5 -I 100 -X 500 --no-discordant --no-mixed*. Sam files containing mapped paired-end reads were converted to bam files, sorted and indexed using samtools. For visualization, the data were normalized and converted to bigWig (bw) format using bamCoverage tool from deepTools with the following arguments: *--normalizeUsing CPM --extendReads 200 --smoothLength 9 -bs 3 -ignore chrM*. Genome browser snapshots containing ChIP-seq data were generated using pyGenomeTracks. ChIP-seq data from public repositories were aligned, and bw files were generated as described above. For SOX2, OCT4, and NANOG ChIP-seq data, peaks were called using MACS2 with q value cutoff of 1e-4.
ChIP-seq heatmaps were generated using computeMatrix and plotHeatmap tools from deepTools.

For quantification of motif frequency within ATAC-seq peaks, we extracted genomic sequences in 200 bp (+/-100 bp) regions around peak summits using bedtools [Quinlan, 2010] and used the resulting fasta files as input for Fimo (version 5.1.1) in the MEME suite [Bailey, 2009] using the q-value 5e-4.
Distribution of peaks based on distances from the nearest promoters based on peak summit and TSS coordinates was calculated using bedtools.

GO term analysis

GO term biological process enrichment analyses were performed using the PANTHER classification system [Mi, 2019] (version 14.0). p-value cut off to select enriched GO terms was set at 1e-5.
T-test
Statistical significance of qRT-PCR as well as ATAC-seq and ChIP-seq density differences was assessed using two-tailed t-test as indicated in figure legends.

Additional references for datasets and software cited in the Key Resources Table.
(Bailey et al., 2009; Buenrostro et al., 2013; De Hoon et al., 2004; George et al., 2007; Kim et al., 2019; Koch et al., 2017; Langmead et al., 2009; Li et al., 2009; Martin, 2011; Mi et al., 2019; Quinlan and Hall, 2010; Ramirez et al., 2016; Ramirez et al., 2018; Saldanha, 2004; Stark and Brown, 2011; Trapnell et al., 2010; Tsaytler et al., 2023; Warnes et al., 2016; White et al., 2013; Wickmam, 2016; Zhang et al., 2008).

RESOURCES TABLE

REAGENT or RESOURCE	SOURCE	IDENTIFIER
Antibodies		
SOX2	R&D Systems	Cat#:AF2018; RRID: AB_355110
OCT4	Santa Cruz	Cat#:sc-8628; RRID: AB_653551
NANOG	Abcam	Cat#:ab80892; RRID: AB_2150114
NANOG	Bethyl Laboratories	Cat#:A300-397A;

		RRID: AB_386108
GAPDH	Cell Signaling	Cat#:5174; RRID: AB_10622025
Bacterial and virus strains		
Biological samples		
Chemicals, peptides, and recombinant proteins		
LIF	Chemicon	ESG1107
Retinoic acid	Sigma-Aldrich	R2625
dTAG13	Tocris	6605
Critical commercial assays		
ScriptSeq Complete low input RNA-Seq kit	Illumina	SLC24H
Deposited data		
ATAC_DMSO_12h	This study	GEO: GSM7696207/ GSM7696208
ATAC_DMSO_24h	This study	GEO: GSM7696209/ GSM7696210
ATAC_dTAG13_12h	This study	GEO: GSM7696211/ GSM7696212
ATAC_dTAG13_24h	This study	GEO: GSM7696213/ GSM7696214
ATAC_RA_2h_DMSO	This study	GEO: GSM7696215
ATAC_RA_6h_DMSO	This study	GEO: GSM7696216
ATAC_RA_12h_DMSO	This study	GEO: GSM7696217
ATAC_RA_24h_DMSO	This study	GEO: GSM7696218
ATAC_RA_48h_DMSO	This study	GEO: GSM7696219
ATAC_RA_2h_dTAG13	This study	GEO: GSM7696220
ATAC_RA_6h_dTAG13	This study	GEO: GSM7696221
ATAC_RA_12h_dTAG13	This study	GEO: GSM7696222
ATAC_RA_24h_dTAG13	This study	GEO: GSM7696223
ATAC_RA_48h_dTAG13	This study	GEO: GSM7696224
RNA_UT	This study	GEO: GSM7696225/ GSM8069022
RNA_RA_3h_DMSO	This study	GEO: GSM7696226/ GSM8069023
RNA_RA_6h_DMSO	This study	GEO: GSM7696227/ GSM8069024
RNA_RA_12h_DMSO	This study	GEO: GSM7696228/ GSM8069025
RNA_RA_24h_DMSO	This study	GEO: GSM7696229/ GSM8069026
RNA_RA_3h_dTAG13	This study	GEO: GSM7696230/ GSM8069027
RNA_RA_6h_dTAG13	This study	GEO: GSM7696231/ GSM8069028
RNA_RA_12h_dTAG13	This study	GEO: GSM7696232/ GSM8069029
RNA_RA_24h_dTAG13	This study	GEO: GSM7696233/ GSM8069030
ChIP_Oct4_RA_6h_DMSO	This study	GEO: GSM7913046
ChIP_Oct4_RA_6h_dTAG13	This study	GEO: GSM7913047
ChIP_Nanog_RA_6h_DMSO	This study	GEO: GSM7913044
ChIP_Nanog_RA_6h_dTAG13	This study	GEO: GSM7913045
mESC WT ATAC-seq	Tsaytler et al., 2023	GEO: GSM5149213/ GSM5149214
mESC ME diff. day 1 WT ATAC-seq	Tsaytler et al., 2023	GEO: GSM5149215/

		GSM5149216
mESC ME diff. day 2 WT ATAC-seq	Tsaytler et al., 2023	GEO: GSM5149217/ GSM5149218
mESC ME diff. day 3 WT ATAC-seq	Tsaytler et al., 2023	GEO: GSM5149219/ GSM5149220
mESC ME diff. day 1 Smad4 WT ATAC-seq	Tsaytler et al., 2023	GEO: GSM5149229/ GSM5149230
mESC ME diff. day 1 Smad4 KO ATAC-seq	Tsaytler et al., 2023	GEO: GSM5149231/ GSM5149232
mESC ME diff. day 2 Smad4 WT ATAC-seq	Tsaytler et al., 2023	GEO: GSM5149233/ GSM5149234
mESC ME diff. day 2 Smad4 KO ATAC-seq	Tsaytler et al., 2023	GEO: GSM5149235/ GSM5149236
mESC ME diff. day 2 Smad4 WT RNA-seq	Tsaytler et al., 2023	GEO: GSM5149211
mESC ME diff. day 2 Smad4 KO RNA-seq	Tsaytler et al., 2023	GEO: GSM5149212
mESC ME diff. day 2 pSmad1/5/9 ChIP-seq	Tsaytler et al., 2023	GEO: GSM5149190
mESC ME diff. day 2 Smad2 ChIP-seq	Tsaytler et al., 2023	GEO: GSM6663202
mESC ME diff. day 2 Eomes ChIP-seq	Tsaytler et al., 2023	GEO: GSM5149192
mESC ME diff. day 3 T ChIP-seq	Tsaytler et al., 2023	GEO: GSM5149194
Mouse reference genome mm10	Genome Reference Consortium	http://hgdownload.soe.ucsc.edu/goldenPath/mm10/
Mouse mm10 RefSeq annotation	NCBI RefSeq project	http://hgdownload.soe.ucsc.edu/goldenPath/mm10/
mESC Oct4 ChIP-seq	Whyte et al., 2013	GEO: GSM1082340
mESC Sox2 ChIP-seq	Whyte et al., 2013	GEO: GSM1082341
mESC Nanog ChIP-seq	Whyte et al., 2013	GEO: GSM1082342
mESC H3K4me1 ChIP-seq	Zhang et al., 2020	GEO: GSM4303796/ GSM4303797
mESC H3K27Ac ChIP-seq	Zhang et al., 2020	GEO: GSM4205678/ GSM4205679
mESC Sox2 ChIP-seq	Lodato et al., 2013	GEO: GSM1050291
mouse NPC Sox2 ChIP-seq	Lodato et al., 2013	GEO: GSM1050288
mouse NPC Brn2 ChIP-seq	Lodato et al., 2013	GEO: GSM1050286
mouse NSC Sox2 ChIP-seq	Engelen et al., 2011	GEO: ERR015312
mouse NPC Sox3 ChIP-seq	Bergsland et al., 2011	GEO: GSM818936
mouse NSC Oct6 ChIP-seq	Mistri et al., 2015	GEO: GSM1711442
mouse NPC Sox1 CUT&RUN	Wen et al., 2023	GEO: GSM6697000
mouse NPC Pax6 CUT&RUN	Wen et al., 2023	GEO: GSM6696988
mESC (Sox2ON) Oct4 ChIP-seq	Friman et al., 2019	GEO: GSM3963087/ GSM3963089
mESC (Sox2OFF) Oct4 ChIP-seq	Friman et al., 2019	GEO: GSM3963088/ GSM3963090
mESC (Sox2ON, 26h) ATAC-seq	Friman et al., 2019	GEO: GSM3961031/ GSM3961034
mESC (Sox2OFF, 26h) ATAC-seq	Friman et al., 2019	GEO: GSM3961032/ GSM3961035
mESC (Sox2ON, 40h) ATAC-seq	Friman et al., 2019	GEO: GSM3961037/ GSM3961040
mESC (Sox2OFF, 40h) ATAC-seq	Friman et al., 2019	GEO: GSM3961038/ GSM3961041
mESC FLC D3 Dox ATAC-seq	Blassberg et al., 2022	GEO: GSM4959456
mESC FLC D3 ATAC-seq	Blassberg et al., 2022	GEO: GSM4959457
mESC Sox2-FKBP_NT ATAC-seq	Maresca et al., 2023	GEO: GSM6373853/ GSM6373854
mESC Sox2-FKBP_6h ATAC-seq	Maresca et al., 2023	GEO: GSM6373861/

		GSM6373862
mESC Sox2-FKBP_24h ATAC-seq	Maresca et al., 2023	GEO: GSM6373863/ GSM6373864
Experimental models: Cell lines		
Mouse F1G4	George et al., 2007	N/A
Mouse Sox2 KO	This paper	N/A
Experimental models: Organisms/strains		
Oligonucleotides		
PCR primers	Star Methods	N/A
Recombinant DNA		
Software and algorithms		
FastQC	Babraham Bioinformatics	https://www.bioinformatics.babraham.ac.uk/projects/fastqc
Cutadapt	Martin M., EMBnet.journal	https://doi.org/10.14806/ej.17.1.200
Hisat2	Kim et al., 2019	https://daehwankimlab.github.io/hisat2
deepTools	Ramirez et al., 2016	https://deeptools.readthedocs.io
samtools	Li et al., 2009	http://www.htslib.org
bedtools	Quinlan et al., 2010	https://github.com/arq5x/bedtools2
Cuffdiff	Trupnell et al., 2010	https://cole-trapnell-lab.github.io/cufflinks/cuffdiff
pyGenomeTracks	Ramirez et al., 2018	https://github.com/deeptools/pyGenomeTracks
PANTHER	Mi et al., 2019	http://www.pantherdb.org
gplots	Warnes et al., 2016	https://CRAN.R-project.org/package=gplots
ggplot2	Wickham, 2016	https://ggplot2.tidyverse.org
R: A Language and Environment for Statistical Computing	R Core Team	https://www.R-project.org
Cluster 3.0	de Hoon et al., 2004	http://bonsai.hgc.jp/~Emdehoon/software/cluster/software.htm
Java TreeView	Saldanha et al., 2004	https://sourceforge.net/projects/jtreeview/
Bowtie	Langmead et al., 2009	http://bowtie-bio.sourceforge.net/index.shtml
MACS2	Zhang et al., 2008	https://github.com/macs3-project/MACS
MEME Suite	Bailey et al., 2009	http://meme-suite.org/
Picard	N/A	https://broadinstitute.github.io/picard/
DiffBind	Stark et al., 2011	https://bioconductor.org/packages/release/bioc/html/DiffBind.html

DESeq2	Love et al., 2014	https://bioconductor.org/packages/release/bioc/html/DESeq2.html
featureCounts	Liao et al., 2014	https://bioconductor.org/packages/release/bioc/html/Rsubread.html
Other		

Supplemental References

Bailey, T.L., Boden, M., Buske, F.A., Frith, M., Grant, C.E., Clementi, L., Ren, J., Li, W.W., and Noble, W.S. (2009). MEME SUITE: tools for motif discovery and searching. Nucleic Acids Res. 37 (Web Server issue), W202-8. 10.1093/nar/gkp335.

Bergsland, M., Ramsköld, D., Zaouter, C., Klum, S., Sandberg, R., and Muhr, J. (2011). Sequentially acting Sox transcription factors in neural lineage development. Genes Dev. 25(23), 2453-64. 10.1101/gad.176008.111

Bibel, M., Richter, J., Lacroix, E., and Barde, Y.-A. (2007). Generation of a defined and uniform population of CNS progenitors and neurons from mouse embryonic stem cells. Nat. Protoc. 2, 1034–1043. https://doi.org/10.1038/nprot.2007.147.

Blassberg, R., Patel, H., Watson, T., Gouti, M., Metzis, V., Delás, M.J., and Briscoe, J. (2022). Sox2 levels regulate the chromatin occupancy of WNT mediators in epiblast progenitors responsible for vertebrate body formation. Nat. Cell Biol. 24(5), 633-644. 10.1038/s41556-022-00910-2.

Buenrostro, J.D., Giresi, P.G., Zaba, L.C., Chang, H.Y., and Greenleaf, W.J. (2013). Transposition of native chromatin for fast and sensitive epigenomic profiling of open chromatin, DNA-binding proteins and nucleosome position. Nat. Methods 10, 1213-1218. 10.1038/nmeth.2688.

De Hoon, M.J., Imoto, S., Nolan, J., and Miyano, N.S. (2004). Open source clustering software. Bioinformatics 20, 1453-1454. 10.1093/bioinformatics/bth078.

Engelen, E., Akinci, U., Bryne, J., Hou, J., Gontan, C., Moen, M., Szumska, D., Kockx, C., van IJcken, W., Dekkers, D.H.W., et al. (2011). Sox2 cooperates with Chd7 to regulate genes that are mutated in human syndromes. Nat. Genet. 43, 607–611. 10.1038/ng.825.

Friman, E.T., Deluz, C., Meireles-Filho, A.C., Govindan, S., Gardeux, V., Deplancke, B., and Suter, D.M. (2019). Dynamic regulation of chromatin accessibility by pluripotency transcription factors across the cell cycle. Elife 8, e50087. 10.7554/eLife.50087.

George, S.H., Gertsenstein, M., Vintersten, K., Korets-Smith, E., Murphy, J., Stevens, M.E., Haigh, J.J., and Nagy, A. (2007). Developmental and adult phenotyping directly from mutant embryonic stem cells. Proc. Natl. Acad. Sci. USA 104(11), 4455-60. 10.1073/pnas.0609277104.

Kim, D., Paggi, J.M., Park, C., Bennett, C., and Salzberg, S.L. (2019). Graph-based genome alignment and genotyping with HISAT2 and HISAT-genotype. Nat. Biotechnol. 37(8), 907-915. doi: 10.1038/s41587-019-0201-4.

Koch, F., Scholze, M., Wittler, L., Schifferl, D., Sudheer, S., Grote, P., Timmermann, B., Macura, K., and Herrmann, B.G. (2017). Antagonistic activities of Sox2 and Brachyury control the fate choice of neuro-mesodermal progenitors. Dev. Cell 42(5), 514-526. 10.1016/j.devcel.2017.07.021.

Langmead, B., Trapnell, C., Pop, M., and Salzberg, S.L. (2009). Ultrafast and memory-efficient alignment of short DNA sequences to the human genome. Genome Biol. 10(3), R25. 10.1186/gb-2009-10-3-r25.

Li, H., Handsaker, B., Wysoker, A., Fennell, T., Ruan, J., Homer, N., Marth, G., Abecasis, G., Durbin, R., and 1000 Genome Project Data Processing Subgroup. (2009). The Sequence Alignment/Map format and SAMtools. Bioinformatics 25(16), 2078-9. 10.1093/bioinformatics/btp352.

Liao, Y., Smyth, G.K., and Shi, W. (2014). featureCounts: an efficient general purpose program for assigning sequence reads to genomic features. Bioinformatics 30(7), 923-930. 10.1093/bioinformatics/btt656.

Lodato, M.A., Ng, C.W., Wamstad, J.A., Cheng, A.W., Thai, K.K., Fraenkel, E., Jaenisch, R., and Boyer, L.A. (2013). SOX2 co-occupies distal enhancer elements with distinct POU factors in ESCs and NPCs to specify cell state. PLoS Genet. 9(2), e1003288. 10.1371/journal.pgen.1003288.

Love, M.I., Huber, W., and Anders, S. (2014). Moderated estimation of fold change and dispersion for RNA-seq data with DESeq2. Genome Biol. 15(12), 550. 10.1186/s13059-014-0550-8.

Maresca, M., van den Brand, T., Li, H., Teunissen, H., Davies, J., and de Wit, E. (2023). Pioneer activity distinguishes activating from non-activating SOX2 binding sites. EMBO J. 42(20), e113150. 10.15252/embj.2022113150.

Mi, H., Muruganujan, A., Huang, X., Ebert, D., Mills, C., Guo, X., and Thomas, P.D. (2019). Protocol Update for large-scale genome and gene function analysis with the PANTHER classification system (v.14.0). Nat. Protoc. 14(3), 703-721. 10.1038/s41596-019-0128-8.

Mistri, T.K., Devasia, A.G., Chu, L.T., Ng, W.P., Halbritter, F., Colby, D., Martynoga, B., Tomlinson, S.R., Chambers, I., Robson, P., and Wohland, T. (2015). Selective influence of Sox2 on POU transcription factor binding in embryonic and neural stem cells. EMBO Rep. 16(9), 1177-91. 10.15252/embr.201540467.

Quinlan, A.R., and Hall, I.M. (2010). BEDTools: a flexible suite of utilities for comparing genomic features. Bioinformatics 26, 841-842. 10.1093/bioinformatics/btq033.

Ramírez, F., Ryan, D.P., Grüning, B., Bhardwaj, V., Kilpert, F., Richter, A.S., Heyne, S., Dündar, F., and Manke, T. (2016). deepTools2: a next generation web server for deep-sequencing data analysis. Nucleic Acids Res. 44(W1), W160-5. 10.1093/nar/gkw257.

Ramírez, F., Bhardwaj, V., Arrigoni, L., Lam, K.C., Grüning, B.A., Villaveces, J., Habermann, B., Akhtar, A., and Manke, T. (2018). High-resolution TADs reveal DNA sequences underlying genome organization in flies. Nat. Commun. 9(1), 189. 10.1038/s41467-017-02525-w.

Saldanha, A.J. (2004). Java Treeview - extensible visualization of microarray data. Bioinformatics 20, 3246-3248. 10.1093/bioinformatics/bth349.

Trapnell, C., Williams, B.A., Pertea, G., Mortazavi, A., Kwan, G., van Baren, M.J., Salzberg, S.L., Wold, B.J., and Pachter, L. (2010). Transcript assembly and quantification by RNA-Seq reveals unannotated transcripts and isoform switching during cell differentiation. Nat. Biotechnol. 28(5), 511-5. 10.1038/nbt.1621.

Tsaytler, P., Liu, J., Blaess, G., Schifferl, D., Veenvliet, J.V., Wittler, L., Timmermann, B., Herrmann, B.G., and Koch, F. (2023). BMP4 triggers regulatory circuits specifying the cardiac mesoderm lineage. Development 150(10), dev201450. 10.1242/dev.201450.

Wen, Q., Zhou, J., Tian, C., Li, X., Song, G., Gao, Y., Sun, Y., Ma, C., Yao, S., Liang, X., et al. (2023). Symmetric inheritance of parental histones contributes to safeguarding the fate of mouse embryonic stem cells during differentiation. Nat. Genet. 55(9), 1555-1566. 10.1038/s41588-023-01477-w.

Whyte, W.A., Orlando, D.A., Hnisz, D., Abraham, B.J., Lin, C.Y., Kagey, M.H., Rahl, P.B., Lee, T.I., and Young, R.A. (2013). Master transcription factors and mediator establish super-enhancers at key cell identity genes. 153(2), 307-19. 10.1016/j.cell.2013.03.035.

Zhang, Y., Liu, T., Meyer, C.A., Eeckhoute, J., Johnson, D.S., Bernstein, B.E., Nusbaum, C., Myers, R.M., Brown, M., Li, W., and Liu, X.S. (2008). Model-based analysis of ChIP-Seq (MACS). Genome Biol. 9(9), R137. 10.1186/gb-2008-9-9-r137.
